# Supplementary material for: Materials informatics for self-assembly of functionalized organic precursors on metal surfaces
Source: Nat Commun. 2018 Jun 25;9:2469. doi: 10.1038/s41467-018-04940-z (PMC6018224; doi:10.1038/s41467-018-04940-z)
Supplement: Supplementary file 1 — Supplementary information [file 41467_2018_4940_MOESM1_ESM.docx]

**Supplementary Note 1. Summary of the GAMMA method**

***Method overview***

The GAMMA method is described in detail in Supplementary Reference 1. With reference to Figure 2 of the main paper, this method considers *N* molecules adsorbed to a perfect crystalline slab of *d* x *d* unit cells, where periodic boundary conditions are imposed on the slab. All atoms in the model are frozen, and the conformation of each molecule is identical. The molecules adsorb to the surface so that their center-of-mass resides over a finite number of points (‘adsorption sites’), and only a finite number of orientations for the molecule are permitted. For a given configuration *σ* of molecules on the surface, the energy of the system is given by

$E\left(\sigma\right)=\sum_{k=1}^{N} u\left(z_{k}\right)+\sum_{i>j}^{N} v\left(z_{i},z_{j}\right)$, (1)

where *u*(*z_k_*) is the energy of the interaction between molecule *z_k_* and the surface, and *v*(*z_i_*, *z_j_*) is the energy of interaction between molecules *z_i_* and *z_j_*. The equilibrium probability of configuration *σ* appearing on the surface is given by the Boltzmann distribution, namely

$p\left(\sigma\right)\propto\exp\left(-E\left(\sigma\right)/k_{B}T\right)$, (2)

where *k_B_* is the Boltzmann constant and *T* is the temperature. The main assumption in this model is that the conformation of the molecules is determined by the surface-molecule interaction, and receives negligible influence from the presence of other molecules.

For any two molecules *z_i_* and *z_j_*, we define *r*(*z_i_*, *z_j_*) as the minimum of the distances between an atom of *z_i_* and an atom of *z_j_*. An *island* is defined as a collection of molecules such that for any molecule *z_i_* in the island, there is another molecule *z_j_* in the island for which *r*(*z_i_*, *z_j_*) < *M_c_*. Here, *M_c_* is a constant parameter called the cut-off distance. Assuming a sufficiently large cut-off distance, we can re-write the energy of configuration *σ* as

$E\left(\sigma\right)=\sum_{I\in\sigma} E\left(I\right)$, (3)

where the notation *I* ∈ *σ* means that the summation is over the islands in the configuration and *E*(*I*) is the energy of the island, which is defined as on the right-hand side of equation (1). Letting *q* denote a possible combination of islands that could be formed from *N* molecules, the probability that island combination *q* appears at thermodynamic equilibrium is given by

$v\left(q\right)\propto n\left(q\right)\prod_{I\in q} \exp\left(-E\left(I\right)/k_{B}T\right)$, (4)

where *n*(*q*) is the number of distinct ways of arranging the islands of *q* on the surface (using translations and two-fold rotations), and the notation *I* ∈ *q* means that the product is over the islands contained in island combination *q*. Note that this definition of *n*(*q*) assumes that the molecules have two-fold rotational symmetry, which is the case for each type of bianthracene molecule considered here. Given all model parameters, the distribution of island combinations *v*(*q*) can be estimated with a Monte Carlo sampling technique (equivalence class sampling), which is discussed in detail in Supplementary Reference 2.

***Method implementation***

For each type of bianthracene molecule considered here, the GAMMA method was implemented in exactly the same way as described in Supplementary Reference 1. Briefly, an appropriate adsorption configuration was identified by relaxing the gas-phase structures onto a three-layer Cu(111) slab *via* density functional theory calculations. Two-fold rotational symmetry was imposed onto these structures after relaxation, which resulted in very minor changes in the molecule conformations. These conformations were then frozen and scanned above a pristine Cu(111) slab in various orientations, allowing us to identify the low-energy adsorption sites and orientations to use in the calculations. In each type of molecule considered here, the same adsorption sites and orientations to those reported for Br_2_BA, (NH_2_)_2_NA, and (CH_3_)_2_BA from Supplementary Reference 1 were identified (also see Supplementary Figure 1). This procedure also gave the values of the surface-molecule interaction energies *u*(*z_k_*) in equation (1). These values are were the order of about 1.5 – 2.5 eV for each molecule. These large surface-molecule interaction energies support the use of the GAMMA method for each system studied here. The pairwise interaction energies *v*(*z_i_*, *z_j_*) were estimated by the same combination of density functional theory and machine learning reported in Supplementary Reference 1. This approach showed a comparable predictive performance to the calculations reported previously. Finally, *n*(*q*) in equation (4) was estimated using the formula presented in Supplementary Reference 1, which holds under low coverage conditions, and Monte Carlo simulations were performed using simulated annealing with temperatures *T* = 200 K, 210 K, …, 300 K and 1,000,000 simulation steps (calculations for Br_2_BA, (NH_2_)_2_BA, and (CH_3_)_2_BA were ran for 1,200,000, 900,000, and 600,000 steps, respectively). Analyses were performed on data from the final 300,000 steps from each simulation. All calculations were performed on a slab containing *d* x *d* = 50 x 50 unit cells, *N* = 10 molecules, and a cut-off distance *M_c_* of 8 Å.

| 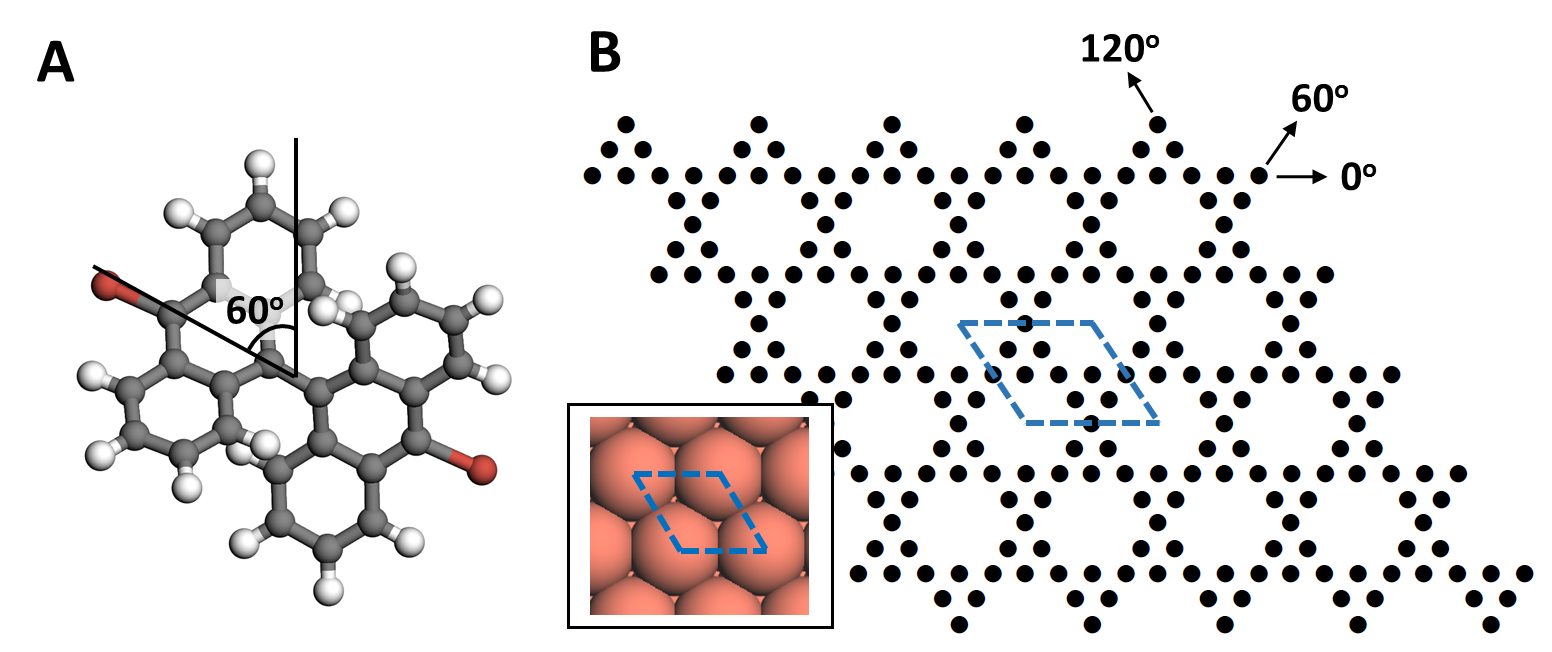  **Supplementary Figure 1**. (A) A single Br2BA molecule adsorbed to a Cu(111) surface. The Cu(111) surface (not explicitly shown) is in the plane of the page. The molecule conformation was chosen by the procedure described under *Model implementation* of Supplementary Note 1. The molecule is in the “60^o^ orientation”, where the orientation angle is defined as the angle between the central C-C bond and the vertical direction. (B) Possible adsorption sites for a single Br_2_BA molecule on a Cu(111) surface, The adsorption sites in the direction of the arrows marked 0^o^, 60^o^, and 120^o^ permit the molecule to take on the 0^o^ 60^o^, and 120^o^ orientations, respectively. The dotted blue lines indicate a single Cu(111) unit cell. The position of the Cu atoms in the Cu(111) unit cell are indicated in the insert. Supplementary Figure 1(B) was adapted from Supplementary reference 3. |
| --- |

| **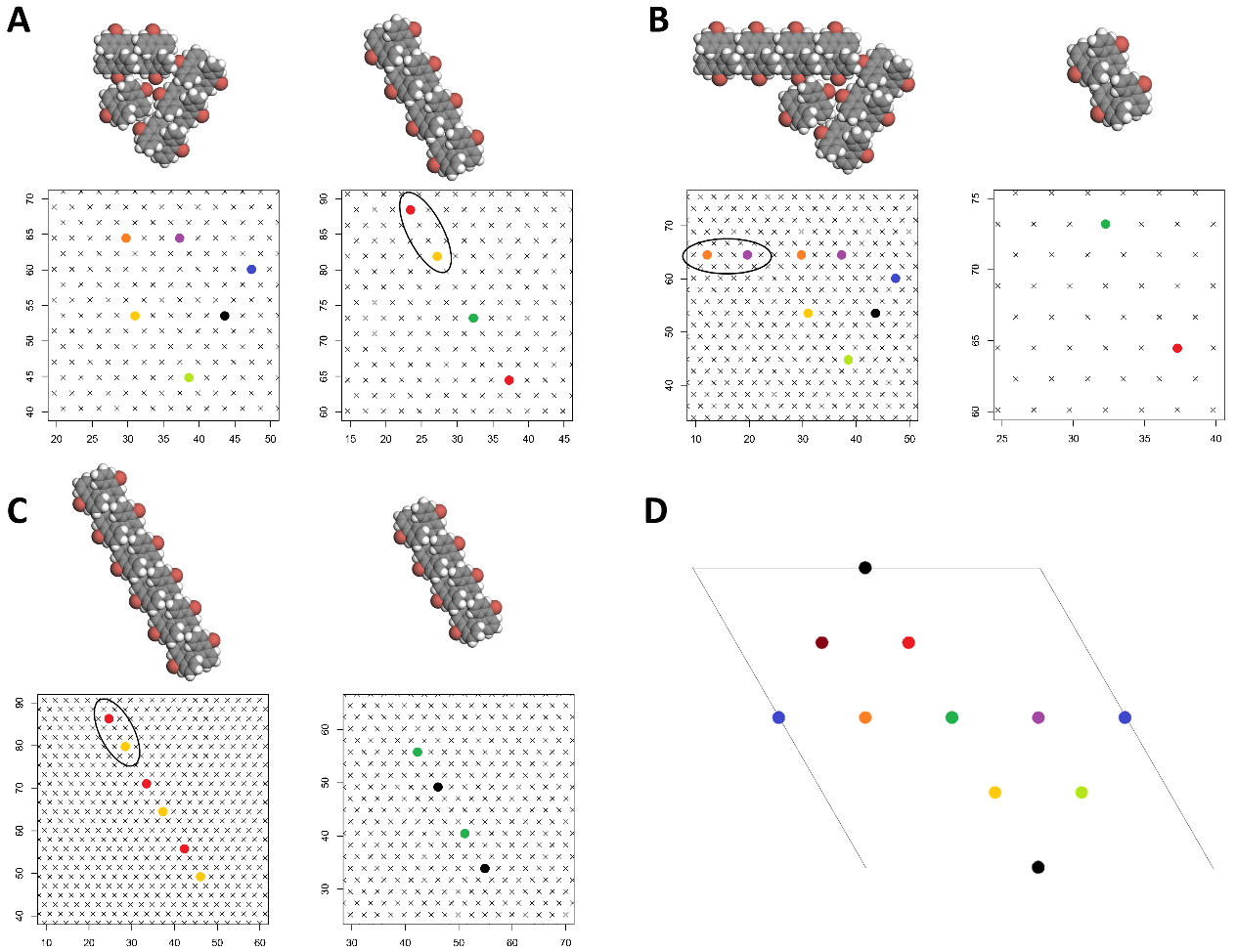**  **Supplementary Figure 2**. This figure demonstrates the calculation of *d*(*q_i_*, *q_j_*)**.** The top parts of A – C show three possible island combinations resulting from Br_2_BA self-assembly on Cu(111). The bottom parts of A – C plot the unit cells of the Cu(111) surface (crosses), and show the unit cells onto which the center-of-mass of the molecules in the islands is projected (colored points). The specific position of the center of mass of each molecule within the unit cell (‘adsorption site’) is indicated by the colors, and the location of the adsorption sites within a single Cu(111) unit cell is shown in Figure D. In these plots, each horizontal and vertical axis is in units of angstroms. Note that these plots are a real-space alternative to the notation used in Figures 2 and 3 of the main text.  Let the island combinations in Figures A, B, and C be denoted by *q*_A_, *q*_B_, and *q*_C_, respectively. Now, in order to transform *q*_A_ into *q*_B_, the two molecules inside the black circle in *q*_A_ must move to the positions indicated by the black circle in *q*_B_. In order to transform *q*_A_ into *q*_B_, the states of two molecules must change. Hence, *d*(*q*_A_, *q*_B_) = 2. In contrast, in order to transform *q*_A_ into *q*_C_, all molecules *except for those in the black circle* in *q*_A_ must be moved. In order to transform *q*_A_ into *q*_C_, the states of eight molecules must change, and hence *d*(*q*_A_, *q*_C_) = 8. |
| --- |

**Supplementary Note 2. Random walk interpretation of *D*(X,Y)**

We interpreted *D*(X,Y) in equation (1) of the main text in terms of a random walk on the island combination network. Separately from this, recall that in our simulations of the GAMMA model, island combinations (or ‘equivalence classes’) are sampled using the Markov chain Monte Carlo (MCMC) method (Supplementary References 1 and 2). In turn, the MCMC method is equivalent to simulating a discrete-time random walk (‘MCMC random walk’) on the space of island combinations. The purpose of this note is to show that the random walk described in the main text can be regarded as the MCMC random walk involved the GAMMA model simulations.

To show this, firstly note that the MCMC random walk used in the GAMMA model simulations evolves by changing the state of the molecules one at a time. The state space of this MCMC random walk is therefore identical to the island combination network shown in Figure 2 of the main text. This implies that *d*(*q_i_*, *q_j_*) is equal to the minimum number of steps that the MCMC random walk must take in order to move from point *q_i_* to point *q_j_*. Secondly, note that this MCMC random walk a special case of the Metropolis-Hastings Markov chain, and is therefore guaranteed to converge to the Boltzmann distribution (equation (2) of the main text) in the long-time limit. The MCMC random walk used in the GAMMA model simulations therefore possesses the essential properties of the random walk described in the main test.

While not physically rigorous, such MCMC random walks are widely used models for long-time behavior of self-assembly and other equilibration processes. We follow this approach in our physical interpretation of *D*(X,Y) in the main text.

**Supplementary Note 3. Short-cut formula for the network distance *d*(*q_i_*, *q_j_*)**

In the dendrogram calculations reported here, we used a short-cut formula to calculate the distance between vertices *q_i_* and *q_j_* in the island combination network without having to generate the network directly. This formula is shown below in Equation (5), however to introduce it several concepts must be defined first.

***Island combinations***

As mentioned above, an ‘island combination’ *q_i_* is defined a set of islands that could be created from the *N* molecules residing on the surface slab. In the following, we must assume that each island combination contains exactly one empty set Ø, where Ø is called the *empty island*. Island combinations *q_i_* and *q_j_* do not differ if *q_i_* can be transformed into *q_j_* by translations or two-fold rotations of its islands (Supplementary Figure 3). The ordering of islands is never considered when distinguishing two island combinations. Note that ‘island combinations’ are referred to as *equivalence classes* in Supplementary References 1 and 2.

***Extension-reduction transformations***

An *extension* *of island* *I* is an island that could result from addition of a single molecule to island *I*. A *reduction* *of island* *I* is an island that could result from deletion of a single molecule from *I*. If *I* contains *N* molecules, then no extensions of *I* exist. Similarly, if *I* = Ø, then no reductions of *I* exist.

Fix an island combination *q_i_*, choose an island *I* from *q_i_*, and then choose another island *J* from *q_i_* such that *J* ≠ Ø and *J* ≠ *I.* Fix another equivalence class *q_j_*. If *q_i_* can be transformed into *q_j_* by extension of island *I* and reduction of island *J*, then *q_i_* and *q_j_* are said to be connected by an *extension-reduction transformation.* If *q_i_* and *q_j_* are connected by an extension-reduction transformation, then we write *φ*(*q_i_*) = *q_j_*.

Note that *φ*(*q_j_*) = *q_i_* whenever *φ*(*q_i_*) = *q_j_.* See Supplementary Reference 2 for further details on the extension-reduction transformation.

***Molecule-exchange network***

Suppose that the number of molecules *N* has been fixed. Let *H* be the set of all unique island combinations that can be created from *N* molecules. As defined in the main text, the *molecule-exchange network* is the graph *G =* (*V, E*), where *V* is the vertex set*, E* is the edge set*, V = H,* and (*q_i_, q_j_*) ∈ *E* if and only if *φ*(*q_i_*) *= q_j_.* The molecule-exchange network is a connected graph (see Supplementary Reference 2) with undirected edges.

***Short-cut formula for d(q_~~i~~_, q_~~j~~_)***

For any two island combinations *q_i_* and *q_j_*, let *d*(*q_i_*, *q_j_*) be the number of edges in the shortest path in *G* connecting *q_i_* with *q_j_*. Set *d*(*q_i_*, *q_j_*) = 0 whenever *q_i_* = *q_j_*. The number *d*(*q_i_*, *q_j_*) is the same as the one that appears in equation (1) of the main text.

Now, let *r* = *q_i_* and *s* = *q_j_* if the number of islands in *q_i_* is less than or equal to *q_j_*, and let *r* = *q_j_* and *s* = *q_i_* otherwise (Supplementary Figure 4). Let *π*(*r*, *s*) be the set of all one-to-one pairings of the islands from *r* with islands from *s*. The short-cut formula for *d*(*q_i_*, *q_j_*) is

$d\left(q_{i},q_{j}\right)=\min_{P\in\pi\left(r,s\right)}Q\left(P\right)$ (5)

where

$Q\left(P\right)=\sum_{\left(I,J\right)\in P} R\left(I,J\right)$, (6)

(*I*, *J*) refers to island *I* from island combination *r* paired with island *J* from island combination *s*, and *R*(*I*, *J*) is the smallest number of molecules that must be removed from island *I* for it to be a subset of island *J*. In this context, a collection of molecules *K* is a subset of island *J* if *K* can be entirely superimposed onto a subset of *J* *via* translations or two-fold rotations alone. Equation (5) allows for the graph distance to be calculated without having to generate any part of the molecule exchange network directly.

***Proof of the short-cut formula for d*(*q_~~i~~_*, *q_~~j~~_*)**

Suppose that *r* = *q_i_* and *s* = *q_j_*, where *r* and *s* are defined as above. Let |*r*| and |*s*| denote the number of islands contained in *r* and *s*, respectively, excluding the empty islands Ø. Now, consider a sequence of *n* extension-reduction transformations *φ* (*r*) = *u*_1_, *φ* (*u*_1_) = *u*_2_, …, *φ* (*u_n_* _- 1_) = *s*. The sequence {*r*, *u*_1_, *u*_2_, …, *u_n_* _– 1_, *s*} can be compactly represented by the notation {*φ ^n^*(*r*) = *s*}. By definition, {*φ ^n^*(*r*) = *s*} is a path in the molecule-exchange network. Because a single application of the extension-reduction transform involves exactly one island reduction, the number of edges *n* in the path {*φ ^n^*(*r*) = *s*} is exactly the number of times that an island from {*I*_1_, …, *I*_|_*_r_*_|_} is reduced as we pass along the path.

Now, consider a one-to-one pairing *P* of each of the islands from *r* with one of the islands from *s*. Without loss, we can consider the case

$P=\left\{\begin{matrix} \left(I_{1},J_{1}\right) \\ \vdots\\ \left(I_{\left|r\right|},J_{\left|r\right|}\right) \end{matrix}\right\}$. (7)

Let *R*(*I_k_*, *J_k_*) be the minimum number of times that island *I_k_* must be reduced to become a subset of island *J_k_*. According to the previous paragraph, we can associate *P* with a path {*φ ^n^*(*r*) = *s*: *n* = *R*(*I*_1_, *J*_1_) + *R*(*I*_2_, *J*_2_) + ⋯ + *R*(*I*_|_*_r_*_|_, *J*_|_*_r_*_|_)}. Finally, let *π*(*r*, *s*) be the set of all one-to-one pairings of each island from *r* with one island from *s*, and let

$P*=\mathop{\mathrm{argmin}}\limits_{P\in\pi\left(r,s\right)} \sum_{\left(I,J\right)\in P} R\left(I,J\right)$. (8)

We can associate *P** with a path such that {*φ ^n^*(*r*) = *s* : *n* = *Q*(*P**)}, where *Q* is defined in equation (6). By definition, this path is the shortest path leading from vertex *r* to vertex *s* (or one of the shortest paths leading from *r* to *s*, if more than one such path happens to exist). This implies that *d*(*r*, *s*) = *Q*(*P**), which is equivalent to equation (5).

| 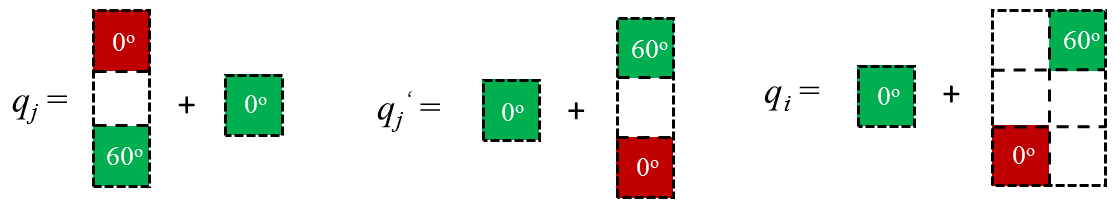  **Supplementary Figure 3**. Three island combinations possible for the case of *N* = 3 molecules adsorbed to the slab (see caption of Figure 2 of the main text for an explanation of the notation). Island combination *q_j_* can be transformed into *q_j_*’ by reordering and rotation of the islands, and so they are equivalent. However, *q_i_* is not equivalent to either of *q_j_* or *q_j_*’. |
| --- |

| 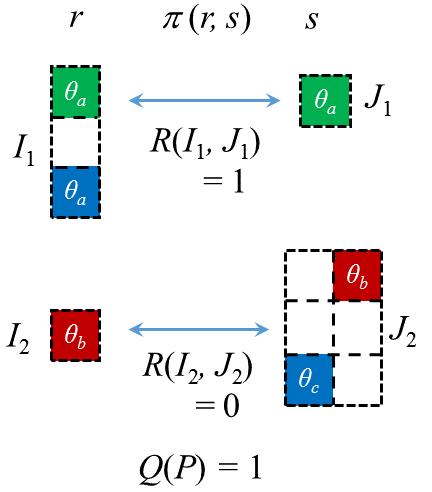  **Supplementary Figure 4**. Diagram to explain equations (5) and (6). Island combination *r* (*s*) corresponds to the two islands on the left (right)-hand side. The blue arrows correspond to *one* pairing from the set π(*r*, *s*). |
| --- |

**Supplementary Note 4. Creation of the dendrogram**

A dendrogram for the case of only three types of bianthracene molecules (X_2_BA, Y_2_BA, and Z_2_BA is shown in Supplementary Figure 5. Assuming that the dissimilarities *D*(X, Y), *D*(X, Z), and *D*(Y, Z) have already been calculated, the dendrogram is created according to the following steps.

Step 1. Place each functional group X, Y, and Z into their own ‘clusters’. The initial clusters are identified by looking at the bottom of the dendrogram in Supplementary Figure 5. For convenience, we denote these initial three clusters as *C*_X_, *C*_Y_, and *C*_Z_, respectively.

Step 2. Identify the two clusters which have the shortest inter-cluster distance. The inter-cluster distance between *C*_X_ and *C*_Y_ is defined as the dissimilarity *D*(X, Y). The inter-cluster distance between *C*_X_ and *C*_Z_, and *C*_Y_ and *C*_Z_, are defined similarly.

Step 3. Suppose that clusters *C*_Y_ and *C*_Z_ are identified as having the shortest inter-cluster distance. *C*_Y_ and *C*_Z_ are then fused to form a new cluster *C*_YZ_. This is identified by the lower of the two horizontal lines in the dendrogram in Supplementary Figure 5. The vertical coordinate of this line is set *D*(Y, Z) = 3.49 (the inter-cluster distance between *C*_Y_ and *C*_Z_).

Step 4. As in steps 2 and 3, we identify the two clusters which have the shortest inter-cluster distance and fuse them together. At this stage, there are only two clusters remaining (*C*_YZ_ and *C*_X_). Because *C*_YZ_ contains more than one functional group, the inter-cluster distance between *C*_YZ_ and *C*_X_ is defined as

max(*D*(X, Y), *D*(X, Z)).

In the present case, the inter-cluster distance works out to be max(*D*(X, Y), *D*(X, Z)) = *D*(Y, Z) = 4.33. *C*_XY_ and *C*_Z_ are fused to form the single cluster *C*_XYZ_. This is identified by the higher of the two horizontal lines in Supplementary Figure 5, which is drawn with vertical coordinate 4.33

In the general case of more than three functional groups, the above scheme is iterated until only a single cluster remains. Whenever we have two clusters *C*_AB…_ and *C*_XY…_containing more than two functional groups, the inter-cluster distance is always defined as the maximum of the distances between a functional group in *C*_AB…_ a functional group in *C*_XY…_. This type of clustering is called *complete linkage.*

| 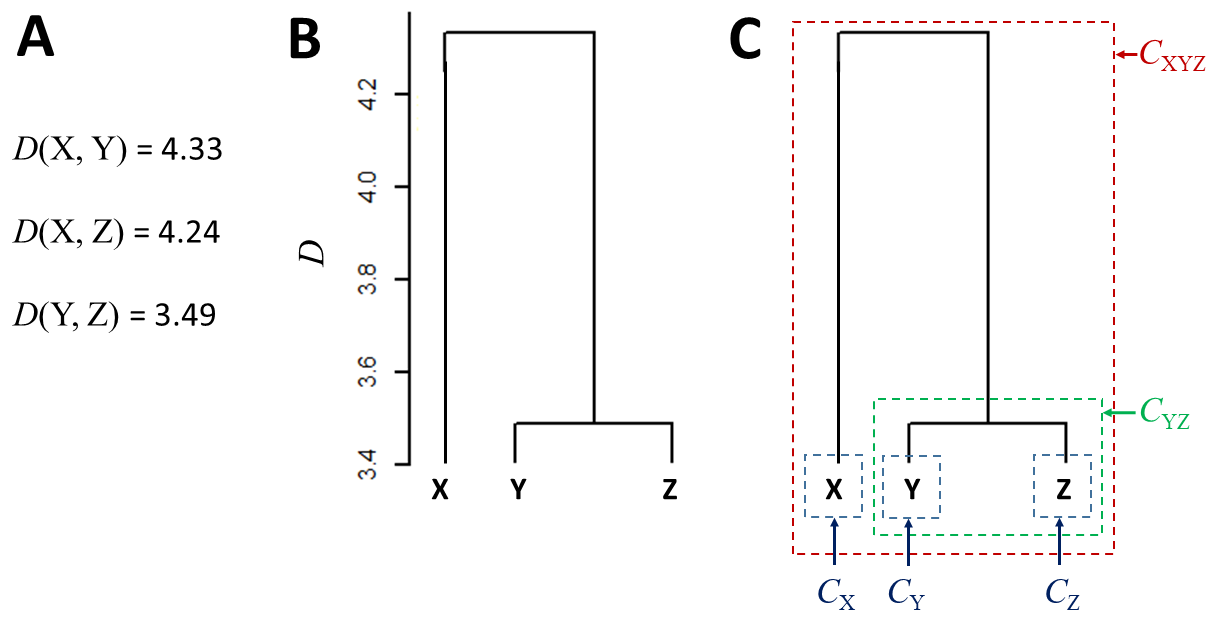 **Supplementary Figure 5**. Diagram to accompany the explanation in Supplementary Note 4. **A** shows the dissimilarities between three functional groups X, Y, and Z. **B** shows the dendrogram constructed from these dissimilarities. **C** shows the same dendrogram in **A**, however the five clusters identified in the dendrogram (*C*_X_, *C*_Y_, *C*_Z_, *C*_YZ_, and *C*_XYZ_) are identified. Note that the boxes in **C** are different from the boxes in Figure 4 of the main paper, which categorize functional groups for the purpose of interpreting the data. |
| --- |


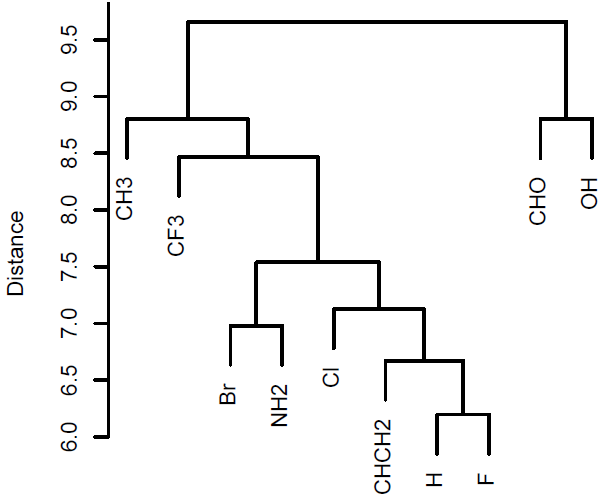


**Supplementary Figure 6.** Dendrogram calculated at 200 K using complete linkage (identical to the dendrogram shown in Figure 4 of the main paper). In complete linkage, the inter-cluster distance between clusters *C*_1_ and *C*_2_ is defined as max *D*(X, Y), where the maximum is taken over all pairs of functional groups X from cluster *C*_1_ and Y from cluster *C*_2_.


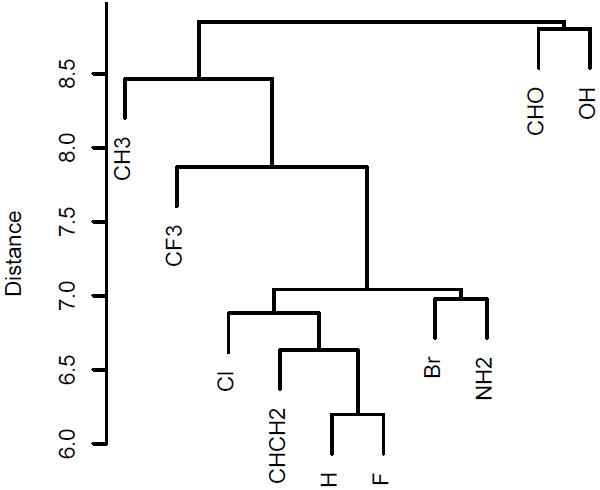


**Supplementary Figure 7.** Dendrogram calculated at 200 K using average linkage. In average linkage, the inter-cluster distance between two clusters *C*_1_ and *C*_2_ is defined as Σ *D*(X, Y)/*n*_1_*n*_2_, where the sum is taken over all pairs of functional groups X from cluster *C*_1_ and Y from cluster *C*_2_, and *n*_1_ and *n*_2_ are the number of functional groups in clusters *C*_1_ and *C*_2_, respectively.


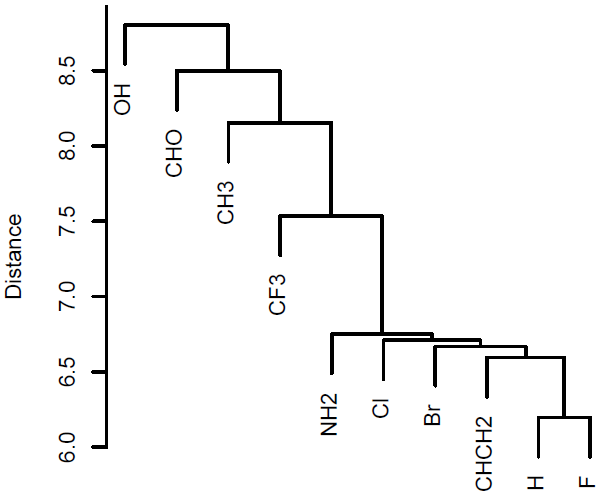


**Supplementary Figure 8.** Dendrogram calculated at 200 K using single linkage. In single linkage, the inter-cluster distance between two clusters *C*_1_ and *C*_2_ is defined as min *D*(X, Y), where the minimum is taken over all pairs of functional groups X from cluster *C*_1_ and Y from cluster *C*_2_.

| **Functionality X** | ***p_C_*** | ***p_NC_*** | ***p_M_*** |
| --- | --- | --- | --- |
| H | 0.96 | 0.04 | 0.00 |
| F | 0.70 | 0.30 | 0.00 |
| CHCH_2_ | 0.55 | 0.39 | 0.06 |
| NH_2_ | 0.43 | 0.52 | 0.05 |
| Cl | 0.835 | 0.155 | 0.01 |
| Br | 0.68 | 0.30 | 0.02 |
| CF_3_ | 0.02 | 0.84 | 0.14 |
| CH_3_ | 0.02 | 0.93 | 0.05 |
| OH | 0.00 | 0.98 | 0.02 |
| CHO | 0.00 | 1.00 | 0.00 |

**Supplementary Table 1.** Probability of seeing chain-shaped (*p_C_*) islands, non-chain-shaped (*p_NC_*) islands, and isolated molecules (*p_M_*) on a 200 K Cu(111) surface, after deposition of X_2_BA molecules and waiting for the system to reach equilibrium. All quantities were estimated from simulations of the GAMMA model, with parameters described in Supporting Note 1. *p_C_* was calculated according to the formula *p_C_* = (1/*M*)Σ*_k_* _= 1, …,_ *_M_ f_k_* , where *f*_1_, …, *f_M_* are each island combinations selected at random from the Monte Carlo sample generated by the simulation, and *f_k_* is the fraction of islands in the island combination which resemble chains, as judged by eye. We used *M* = 100 for each functional group. *p_NC_* and *p_M_* were calculated similarly.

**
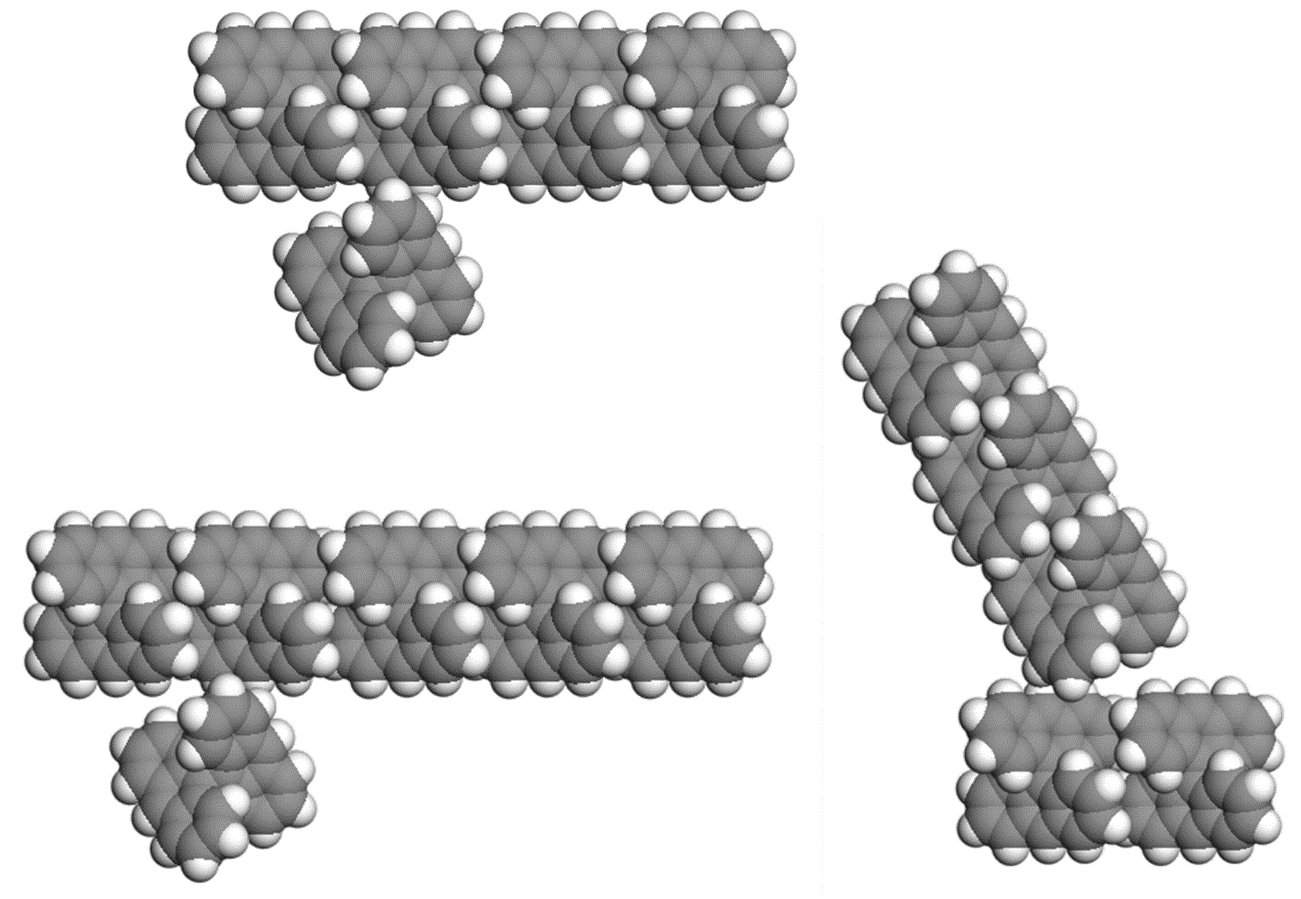
**

**Supplementary Figure 9.** Three typical non-chain-shaped islands resulting from self-assembly of H_2_BA on Cu(111) at 200 K, as predicted by the GAMMA simulations with parameters described above.


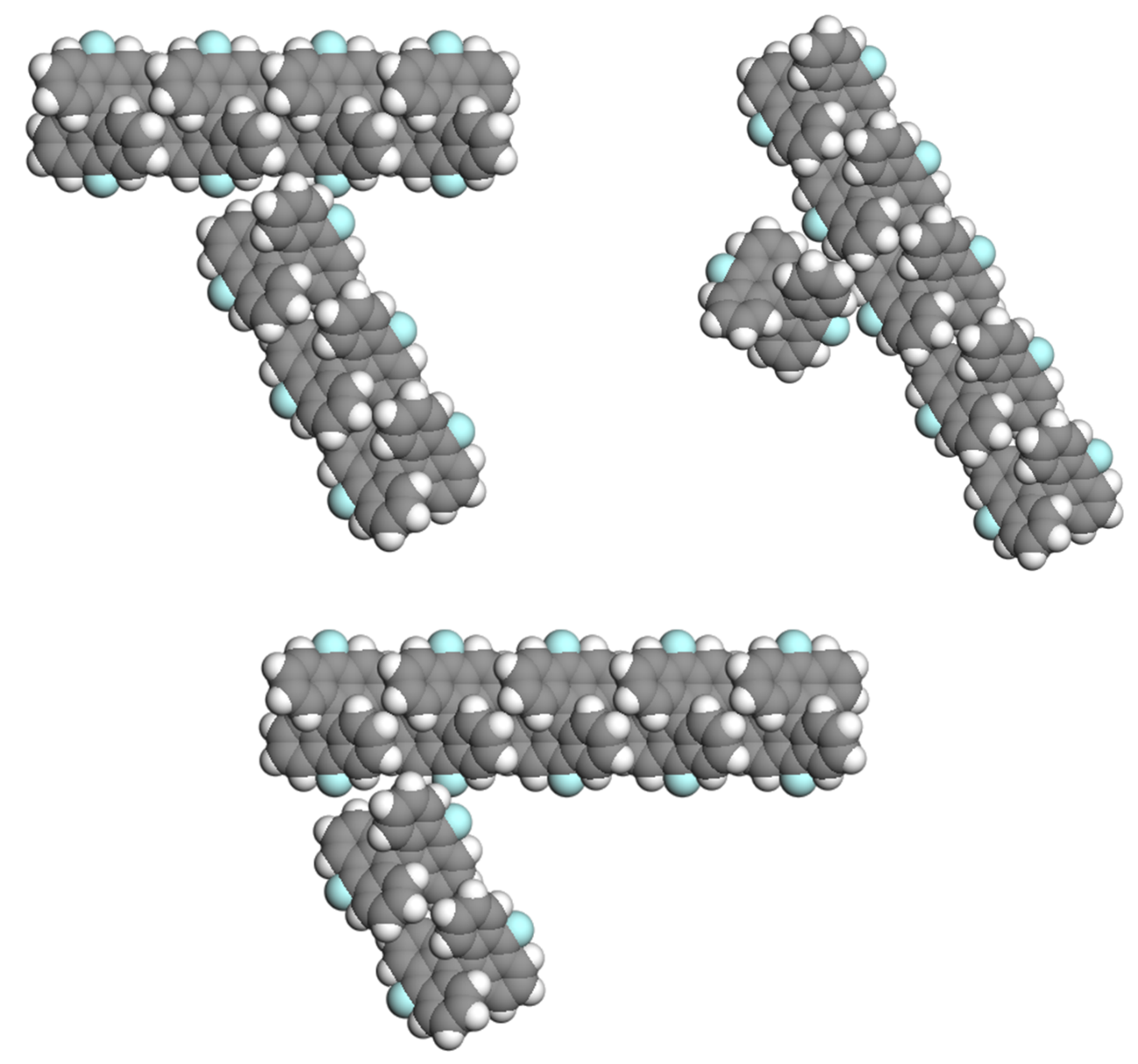


**Supplementary Figure 10.** Three typical non-chain-shaped islands resulting from self-assembly of F_2_BA on Cu(111) at 200 K, as predicted by the GAMMA simulations with parameters described above.


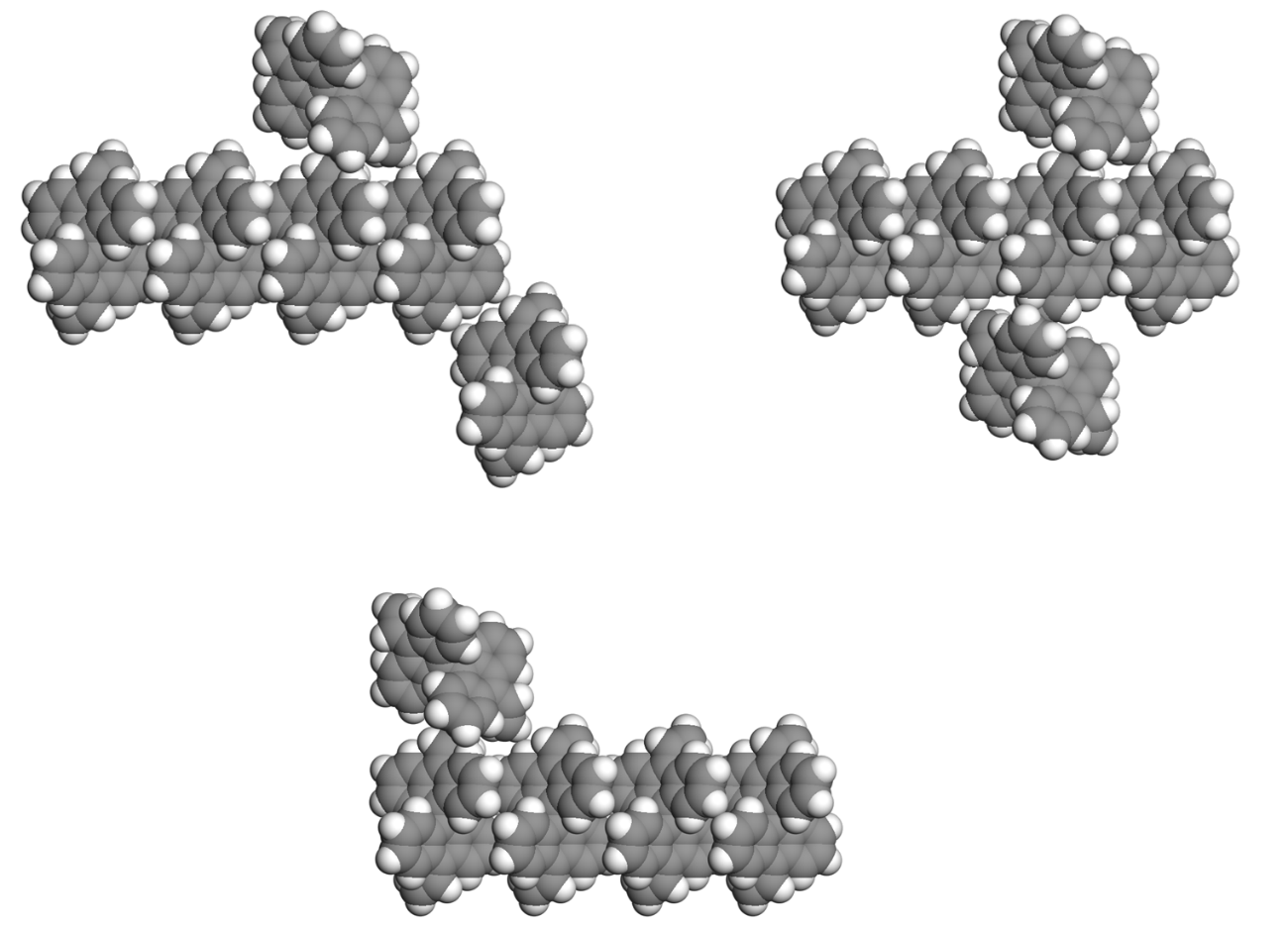


**Supplementary Figure 11.** Three typical non-chain-shaped islands resulting from self-assembly of (CHCH_2_)_2_BA on Cu(111) at 200 K, as predicted by the GAMMA simulations with parameters described above.


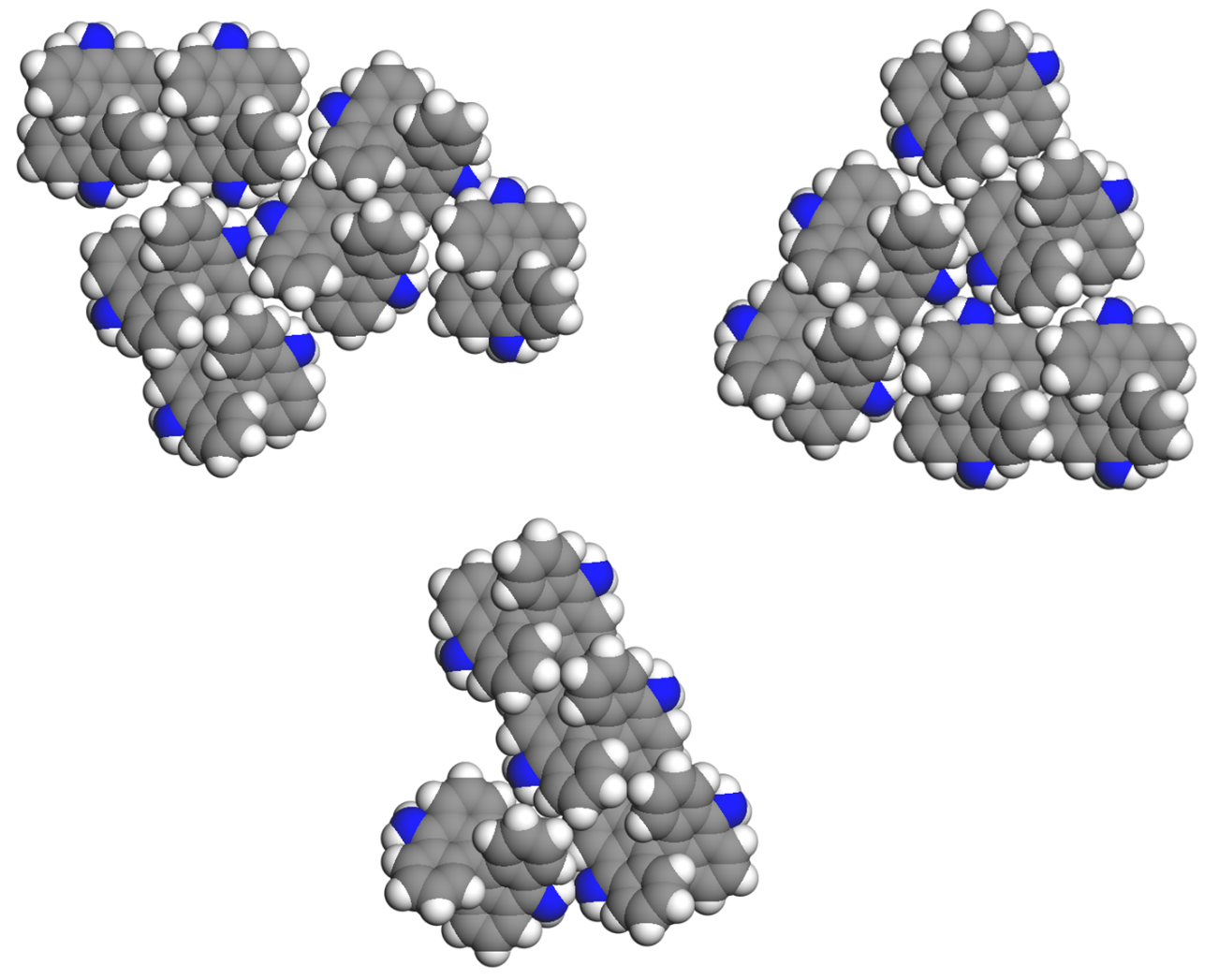


**Supplementary Figure 12.** Three typical non-chain-shaped islands resulting from self-assembly of (NH_2_)_2_BA on Cu(111) at 200 K, as predicted by the GAMMA simulations with parameters described above.


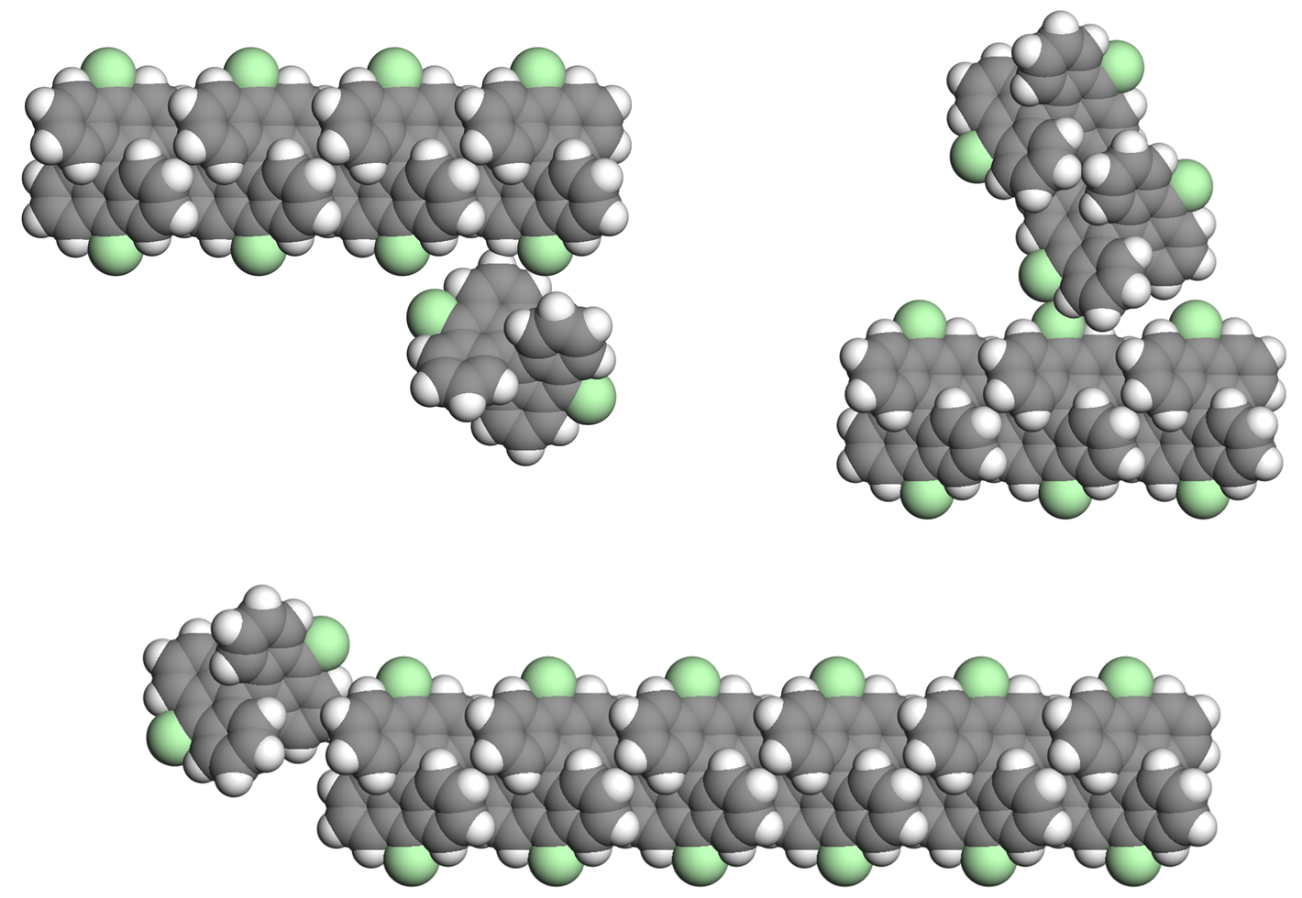


**Supplementary Figure 13.** Three typical non-chain-shaped islands resulting from self-assembly of Cl_2_BA on Cu(111) at 200 K, as predicted by the GAMMA simulations with parameters described above.


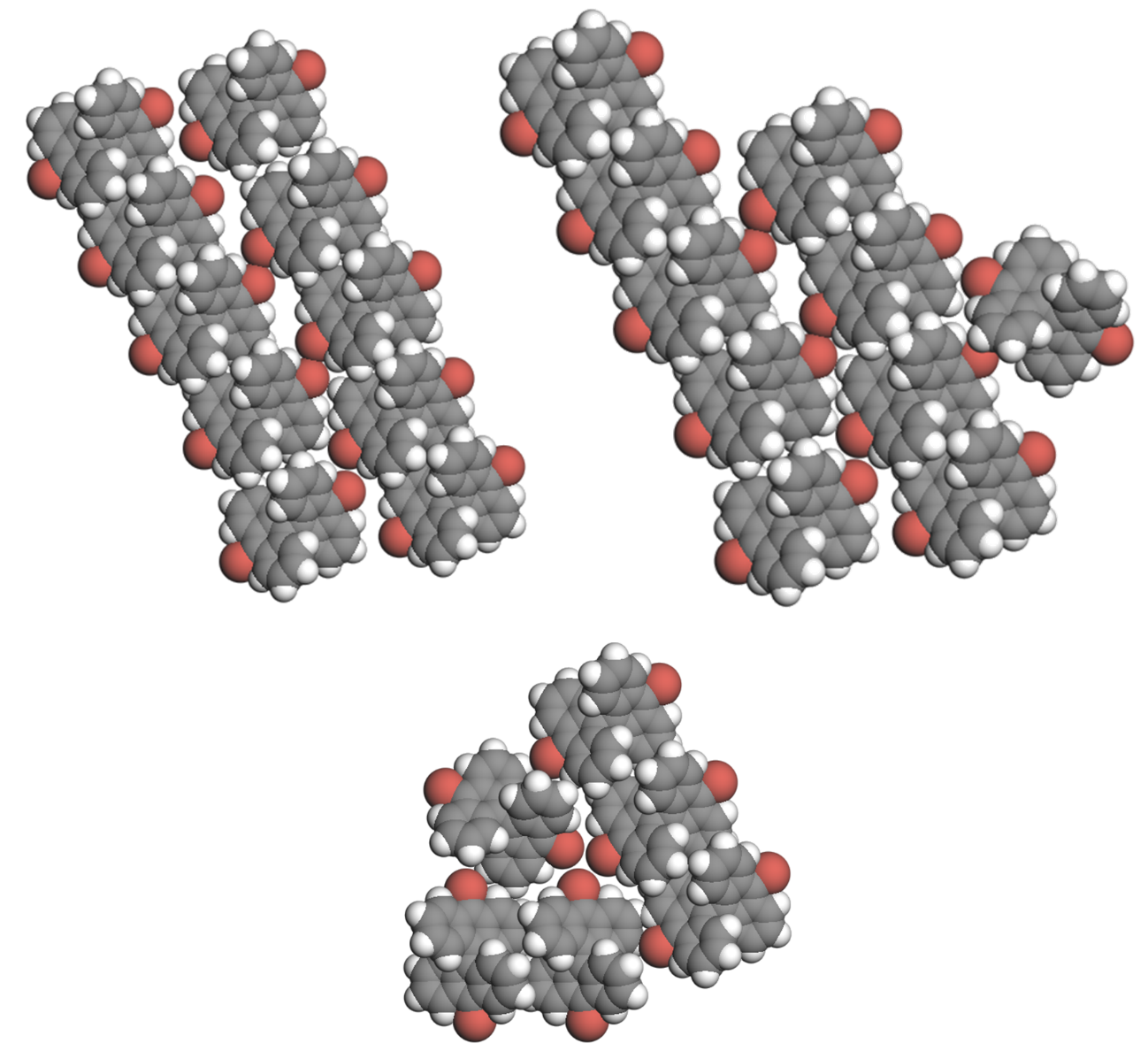


**Supplementary Figure 14.** Three typical non-chain-shaped islands resulting from self-assembly of Br_2_BA on Cu(111) at 200 K, as predicted by the GAMMA simulations with parameters described above.


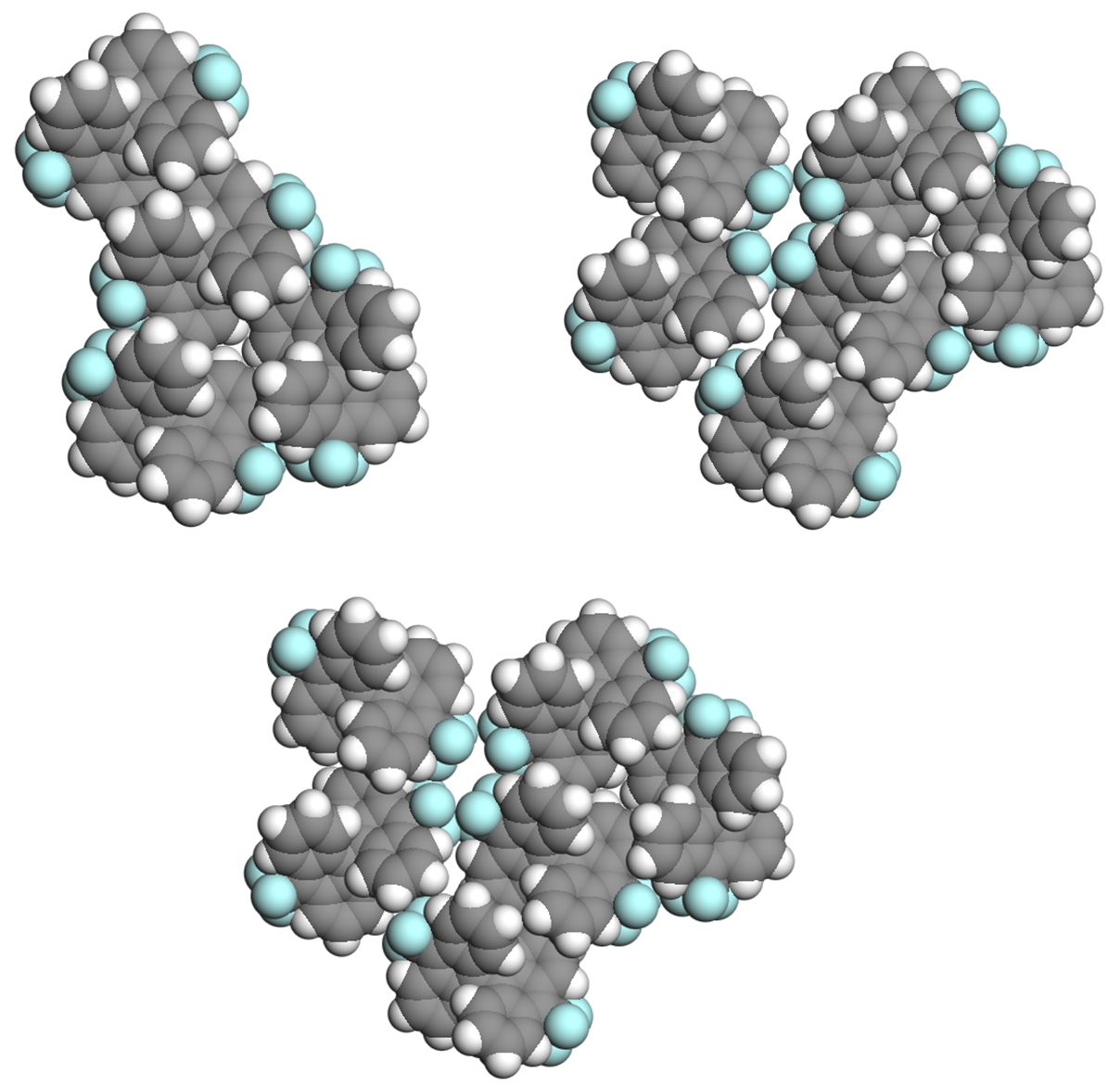


**Supplementary Figure 15.** Three typical non-chain-shaped islands resulting from self-assembly of (CF_3_)_2_BA on Cu(111) at 200 K, as predicted by the GAMMA simulations with parameters described above.


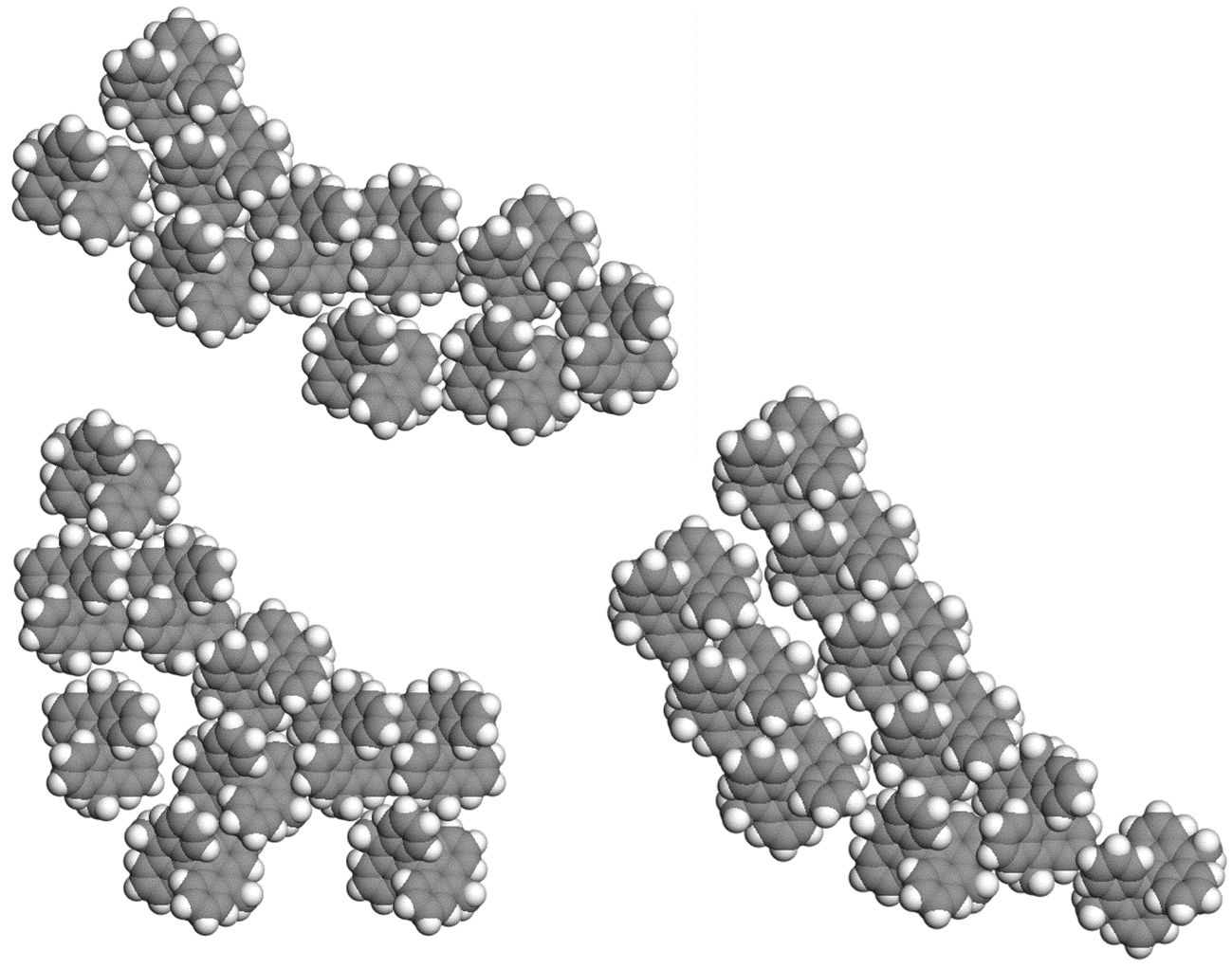


**Supplementary Figure 16.** Three typical non-chain-shaped islands resulting from self-assembly of (CH_3_)_2_BA on Cu(111) at 200 K, as predicted by the GAMMA simulations with parameters described above.


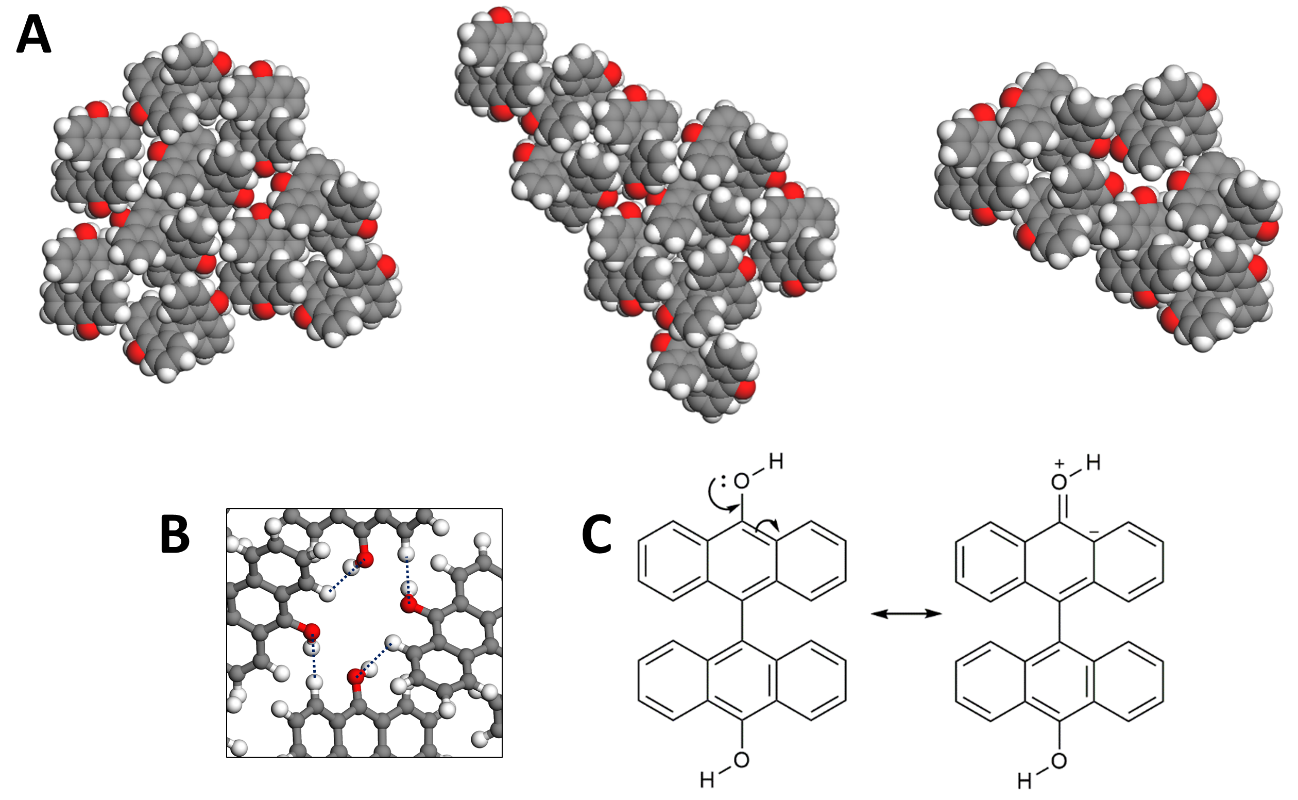


**Supplementary Figure 17.** (A) Three typical non-chain-shaped islands resulting from self-assembly of (OH)_2_BA on Cu(111) at 200 K, as predicted by the GAMMA simulations with parameters described above. The tiling pattern mentioned in the main text results from the ‘three-way star’-type arrangements of adopted by groups of three molecules. (B) Close-up of how how the functional groups of one molecule interact with other molecules. The dotted lines indicate possible hydrogen bonds. (C) Possible resonance structures for the precursor molecule.


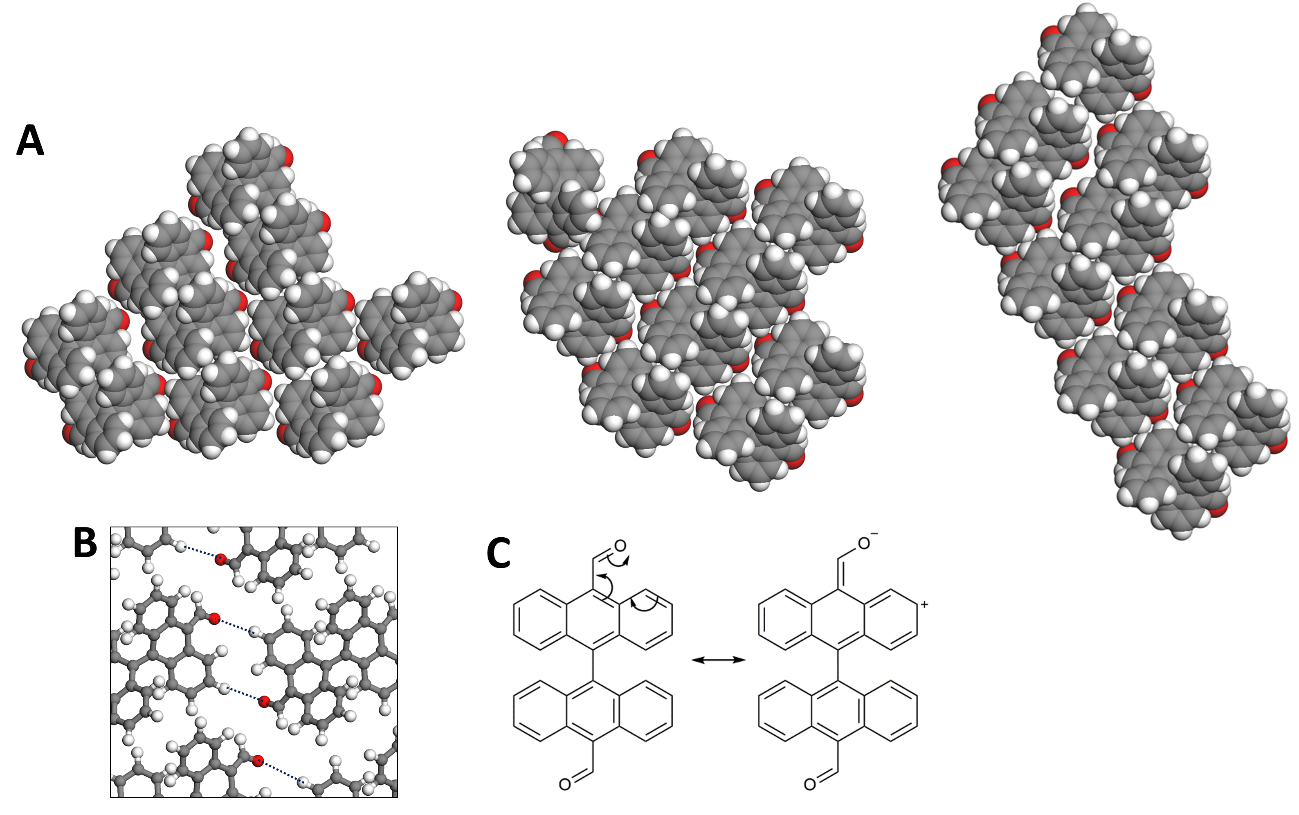


**Supplementary Figure 18.** (A) Three typical non-chain-shaped islands resulting from self-assembly of (CHO)_2_BA on Cu(111) at 200 K, as predicted by the GAMMA simulations with parameters described above. (B) Close-up of how the functional groups of the molecule interact with each other. The dotted lines indicate possible hydrogen bonds. (C) Possible resonance structures for the precursor molecule.

**
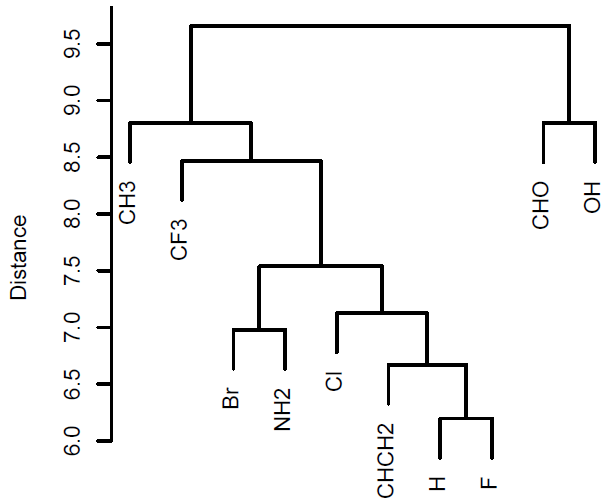
**

**Supplementary Figure 19.** Dendrogram calculated at 200 K for bianthracene precursors on Cu(111) (identical to the dendrogram shown in Figure 4 of the main paper).


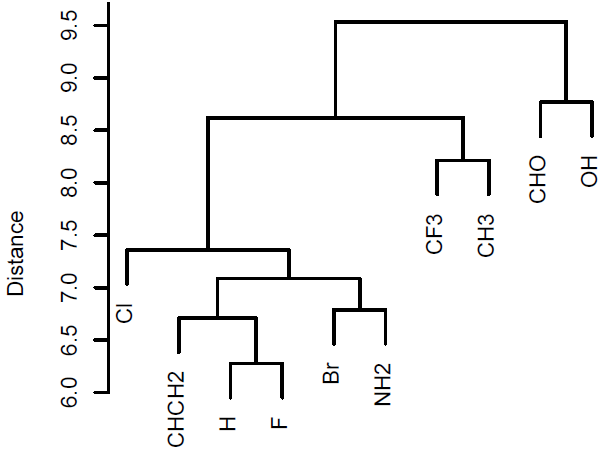


**Supplementary Figure 20.** Dendrogram calculated at 210 K for bianthracene precursors on Cu(111)


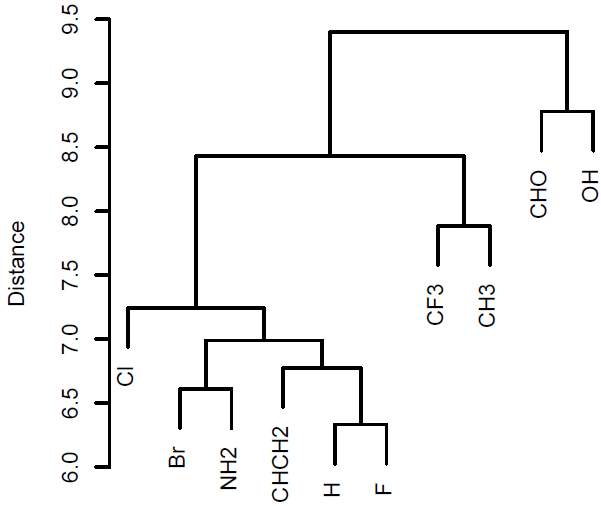


**Supplementary Figure 21.** Dendrogram calculated at 220 K for bianthracene precursors on Cu(111)


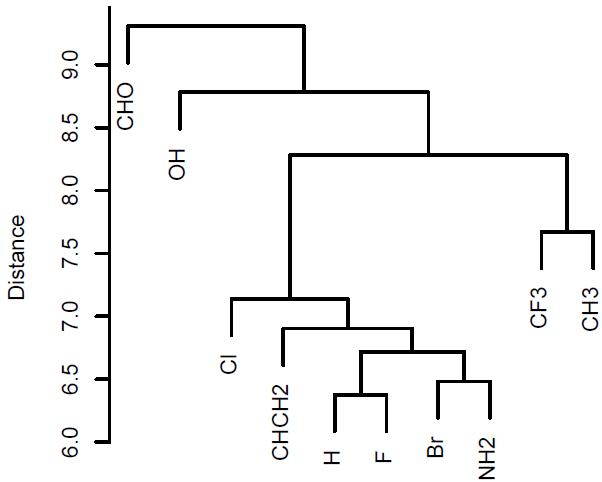


**Supplementary Figure 22.** Dendrogram calculated at 230 K for bianthracene precursors on Cu(111).


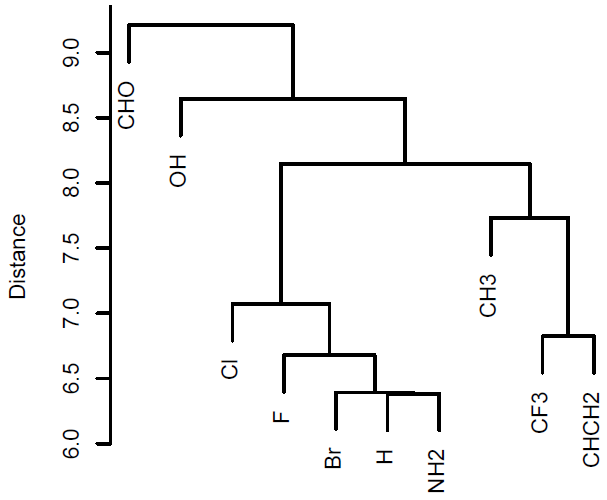


**Supplementary Figure 23.** Dendrogram calculated at 240 K for bianthracene precursors on Cu(111).


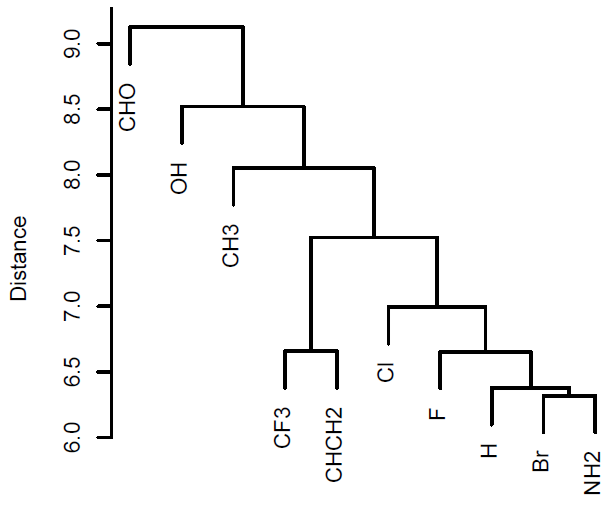


**Supplementary Figure 24.** Dendrogram calculated at 250 K for bianthracene precursors on Cu(111)


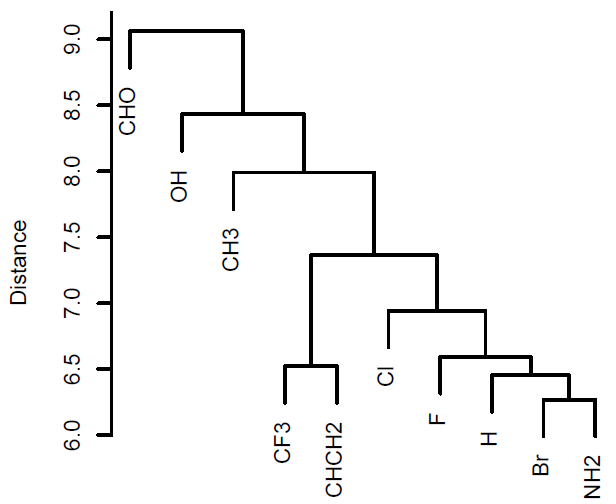


**Supplementary Figure 25.** Dendrogram calculated at 260 K for bianthracene precursors on Cu(111)


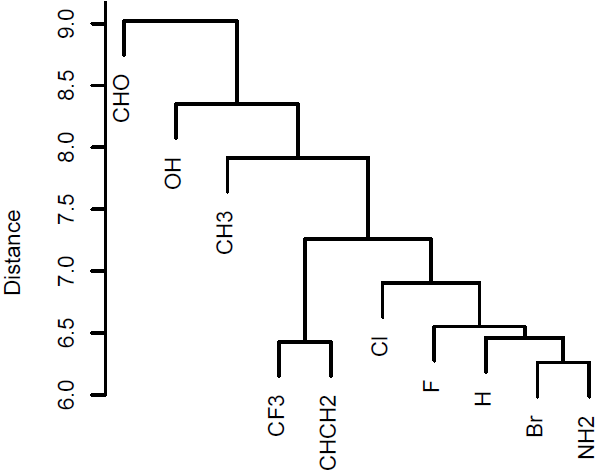


**Supplementary Figure 26.** Dendrogram calculated at 270 K for bianthracene precursors on Cu(111).

**Supplementary Note 5. Application to other surfaces**

In order to demonstrate the generality of our analysis to surfaces other than Cu(111), we constructed the dendrogram for the case of H_2_BA, Br_2_BA, and (CH_3_)_2_BA adsorbed to a Cu(100) surface at 200 K. Because we are mainly interested in showing how the surface affects the island shapes and the dendrogram, we used the same molecule adsorption conformations and pairwise interaction potentials as in the Cu(111) case. Additional DFT calculations show that the relaxed conformation of these molecules on Cu(100) do not differ markedly from their conformations on Cu(111) (Supplementary Figure 27). Moreover, the pairwise interaction potentials mentioned above was trained using intermolecular distances only, and should therefore be transferrable to other types of surfaces.

By scanning the precursor molecules over the Cu(100) surface and calculating the adsorption energy on-the-fly (using the DFT procedure from Supplementary Reference 1), we obtain a set of stable adsorption sites (‘colors’) and orientations for a single bianthracene precursor on Cu(100) (Supplementary Figure 28). By comparing to Supplementary Figure 1B, we see that bianthracene precursors adsorb in different locations and orientations on the Cu(100) unit cell compared to the Cu(111) unit cell. This reflects the strong epitaxial interaction between the bianthracene units and the atomic planes of the copper surface.

By generating island combinations using the GAMMA model (see Supporting Note 1), and computing the dissimilarity *D*(X,Y) in equation (1) for X, Y = H, Br, and CH_3_, we arrive at the dendrogram shown in Supplementary Figure 29. H_2_BA is again observed to form chain-shaped islands with high selectivity (Supporting Table 2), and so H_2_BA on Cu(100) is classified as a strong 1D crystal former. Surprisingly, we find that Br_2_BA on Cu(100) forms chain-shaped islands with even higher selectivity than H_2_BA (Supporting Table 2). By inspection of the island sample generated by the GAMMA model simulation, the chains formed by Br_2_BA tend to be slightly longer than those of H_2_BA, which accounts for the observation that D(H, BR) is around 3.5 in the dendrogram. While we again find that (CH_3_)_2_BA does not form isolated chains on the Cu(100) surface, we find that its islands tend to resemble ‘clusters’ of chains tightly bunched together (Supplementary Figure 32). This differs from the case of the Cu(111) surface, in which the islands from (CH_3_)_2_BA possess far less structural order. While the islands formed by (CH_3_)_2_BA look ‘chain-like’, we find that *D*(CH_3_,H) = 9.61, which is very large. For the case of the ‘chain cluster’-like islands on Cu(100), the interactions between the chains will affect the distances between molecules within a chain, reducing the similarity to the isolated chains produced by the H_2_BA and Br_2_BA precursors. Based on the dendrogram in Supplementary Figure 29, we can identify two categories of bianthracene functional groups for the case of a Cu(100) surface: the strong 1D crystal formers (which contains H and Br), and the weak 2D crystal formers (which contains CH_3_). Additional categories might be identified if a larger number of functional groups are considered.

| **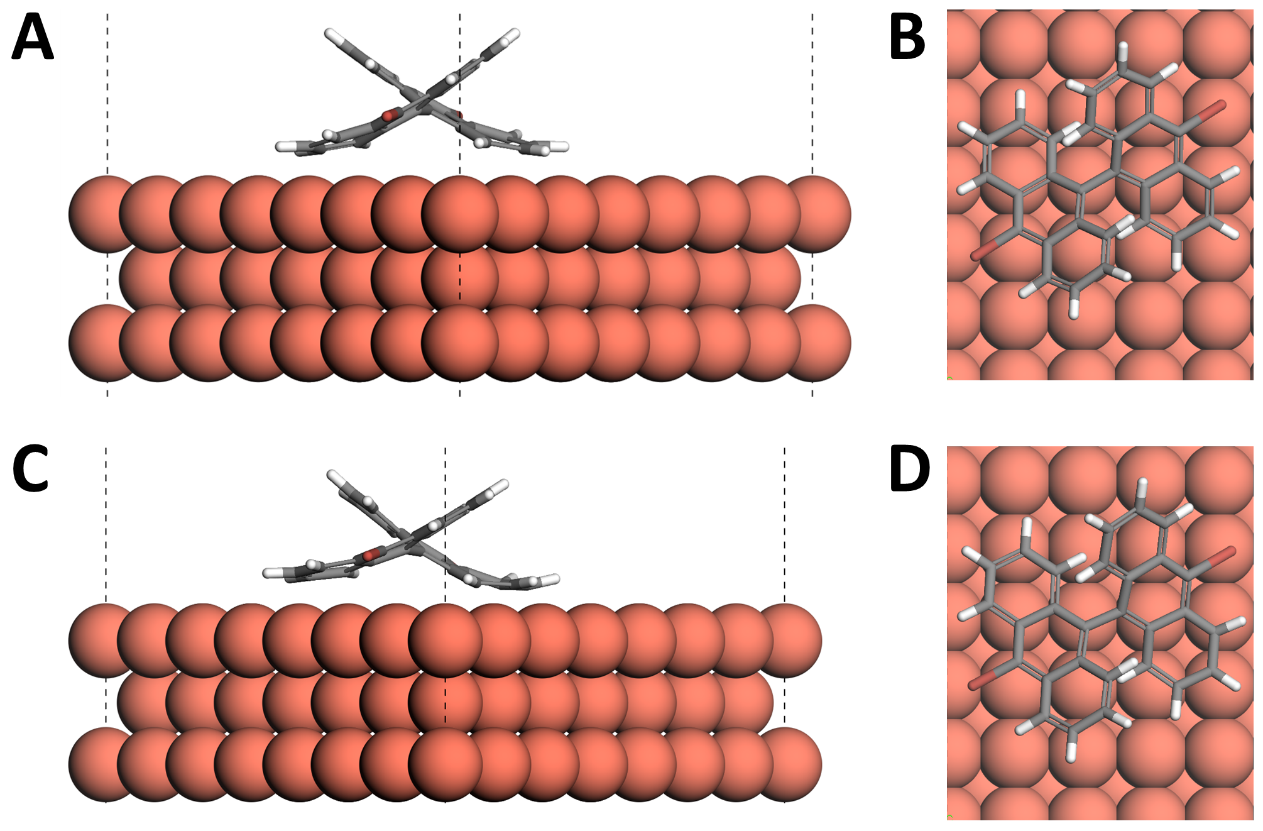**  **Supplementary Figure 27.** (A) Single Br_2_BA molecule adsorbed to a Cu(100) surface, where the conformation of the molecule is identical to the Cu(111)-adsorbed conformation. In this figure, the surface slab has been rotated 45^o^ and oriented perpendicular to the page. The dotted lines indicate the boundaries of the slab. (B) Same as (A), but with the surface slab in the plane of the page. (C, D) are identical to (A) and (B), respectively, but following structural relaxation of the Br_2_BA molecule. The molecule has been drawn as a stick model, so that its conformation can be easily discerned. | | | |
| --- | --- | --- | --- |
| **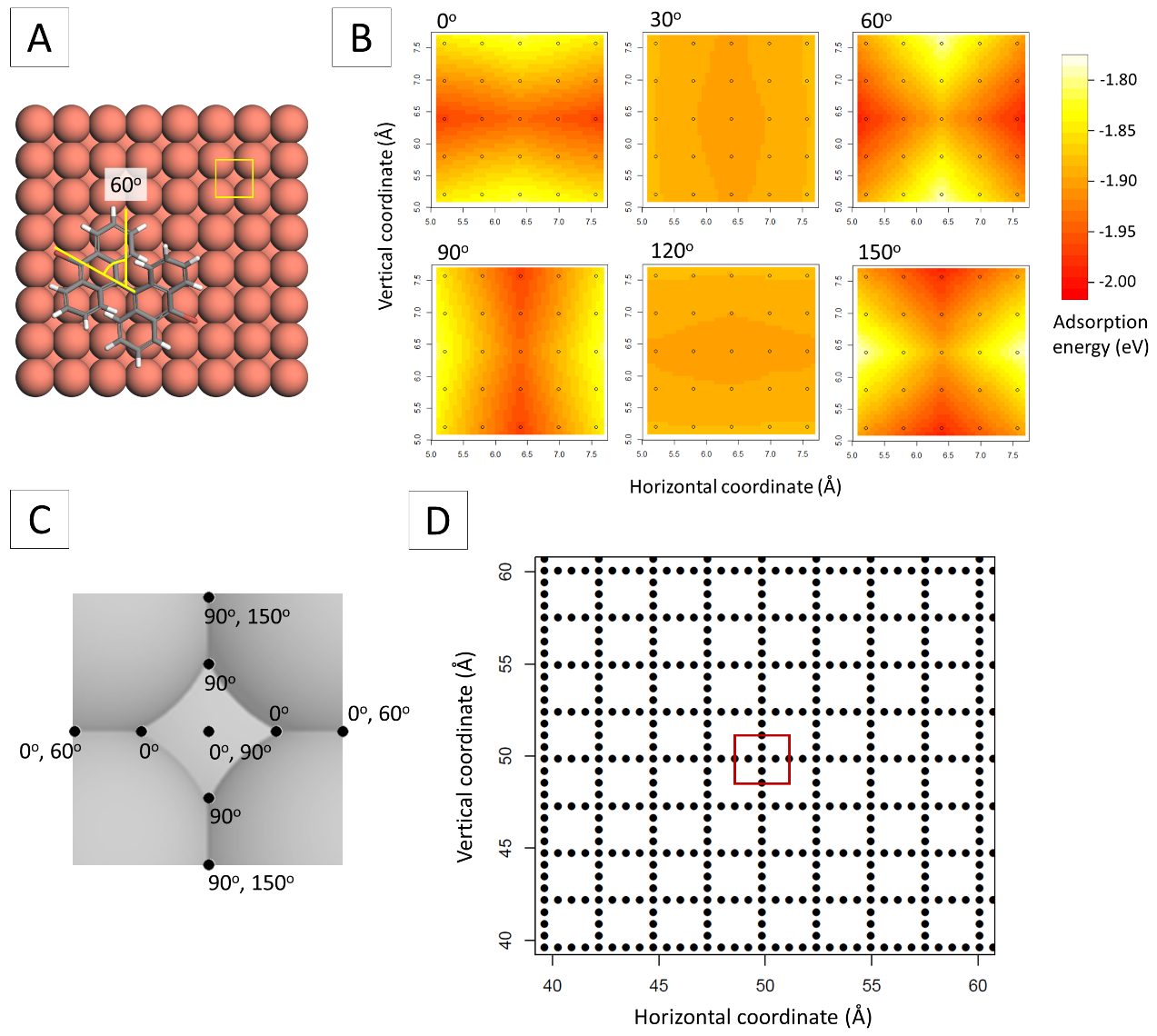**  **Supplementary Figure 28.** (A) A Br_2_BA molecule adsorbed to Cu(100) in the 60^o^ orientation. The orientation is defined as the angle between the vertical axis and the central C-C bond in the molecule, as indicated by the yellow construction lines in the figure. The yellow box indicates a single unit cell of the Cu(100) surface. This molecule has the same conformation as shown in Supplementary Figure 27A and B. (B) Adsorption energy for the molecule at various points within the Cu(100) unit cell, in various orientations. For each point, the adsorption energy is calculated by placing the molecule such that its center-of-mass lies directly above the point, computing the total energy of the system, and then subtracting the energy of the surface alone and the energy of the molecule alone (C) Stable adsorption sites and orientations for the Br2BA molecule within a Cu(100) unit cell. These sites and orientations are identified from the dark red regions from (B). Each adsorption site in (C) corresponds to one possible ‘color’ for the adsorbed molecule. (D) Lattice of adsorption sites obtained by translating the pattern in (C). A single Cu(100) unit cell is indicated by the red square. This figure should be compared to Supplementary Figure 1, which pertains to the case of a Cu(111) surface. | | | |
| **Functionality X** | ***p_C_*** | ***p_NC_*** | ***p_M_*** |
| H | 0.90 | 0.09 | 0.01 |
| Br | 0.95 | 0.04 | 0.01 |
| CH_3_ | 0.01 | 0.94 | 0.05 |

**Supplementary Table 2.** Probability of seeing chain-shaped (*p_C_*) islands, non-chain-shaped (*p_NC_*) islands, and isolated molecules (*p_M_*) on a 200 K Cu(100) surface, after deposition of X_2_BA molecules and waiting for the system to reach equilibrium. See the caption of Supporting Table 1 for additional details.

| **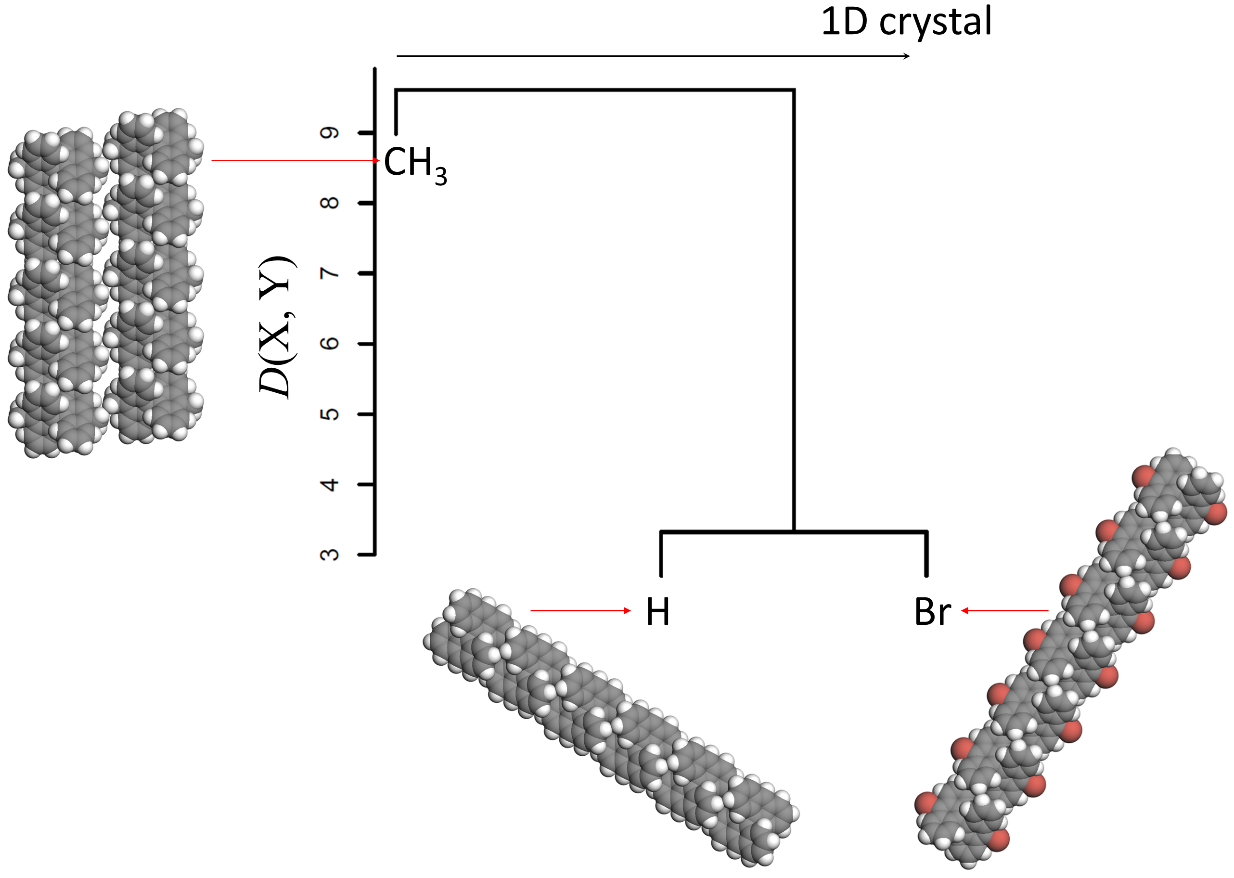**  **Supplementary Figure 29.** Dendrogram constructed for a Cu(100) surface and the bianthracene precursors with functional groups H, Br, and CH_3_. Typical island shapes for each precursor are illustrated. |
| --- |

**
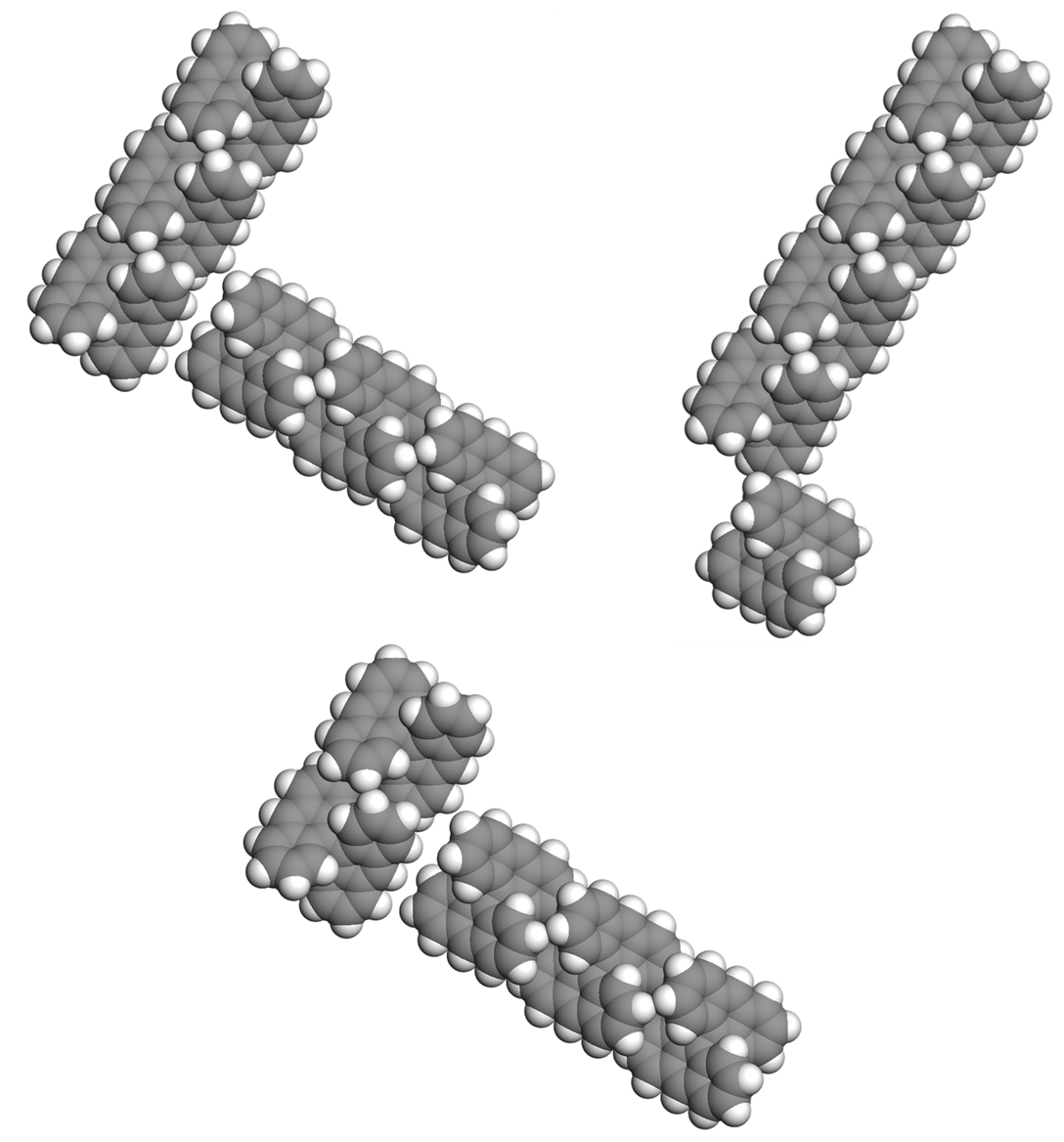
**

**Supplementary Figure 30.** Three typical non-chain-shaped islands resulting from self-assembly of H_2_BA on Cu(100) at 200 K, as predicted by the GAMMA simulations with parameters described above.

**
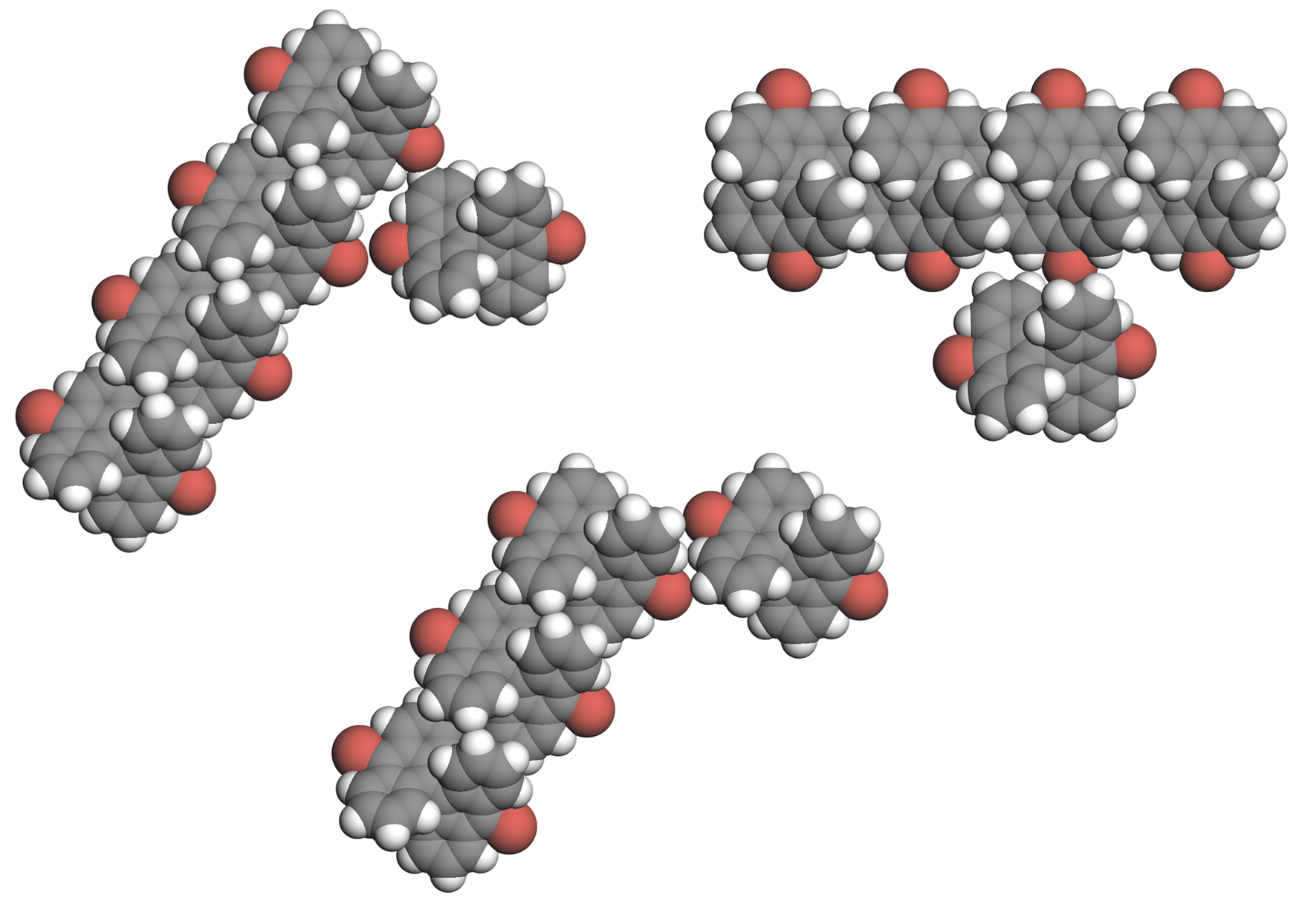
**

**Supplementary Figure 31.** Three typical non-chain-shaped islands resulting from self-assembly of Br_2_BA on Cu(100) at 200 K, as predicted by the GAMMA simulations with parameters described above.

**
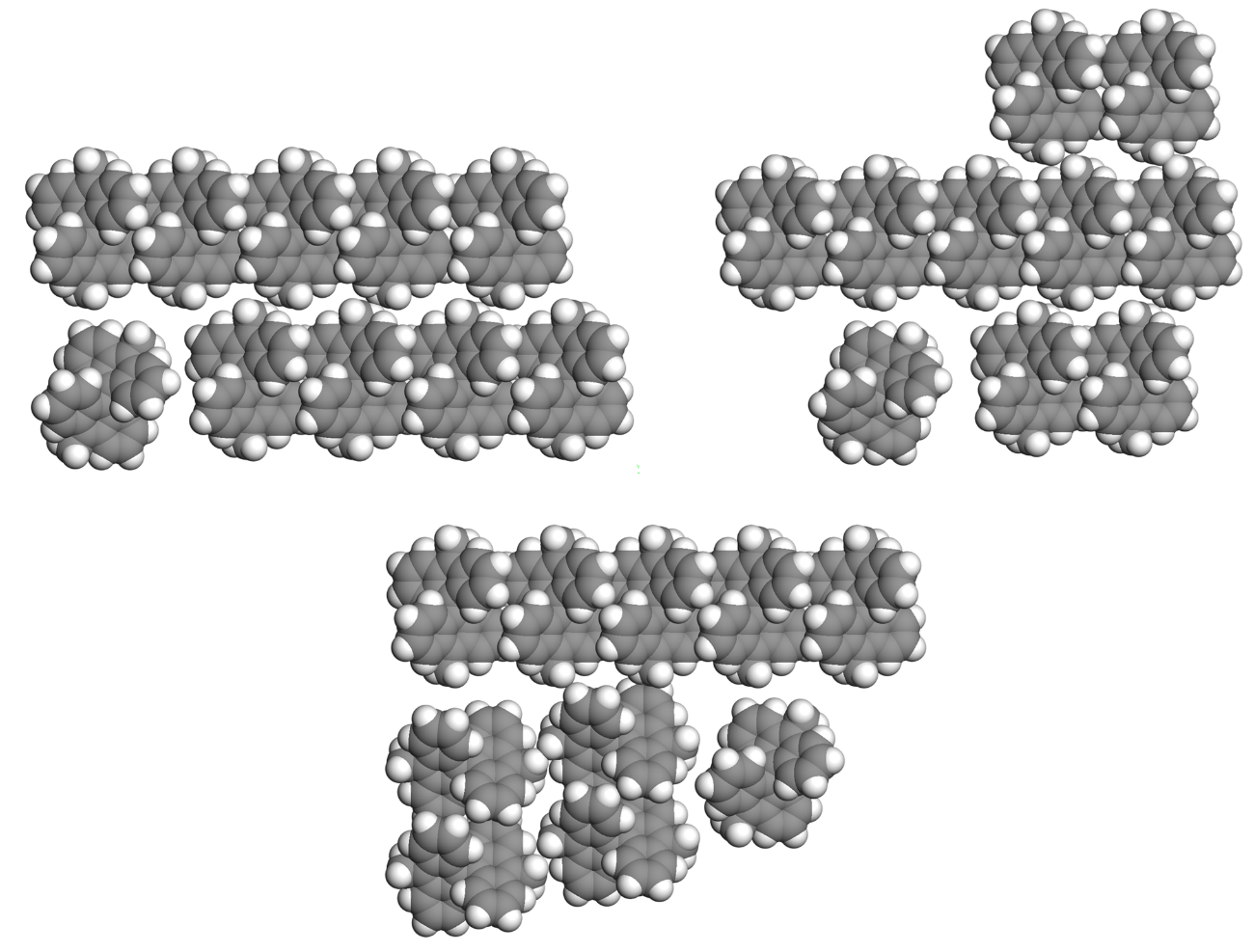
**

**Supplementary Figure 32.** Three typical non-chain-shaped islands resulting from self-assembly of (CH_3_)_2_BA on Cu(100) at 200 K, as predicted by the GAMMA simulations with parameters described above.

**Supplementary References**

1. Packwood, D. M., Han, P., & Hitosugi, T. Chemical and entropic control on the molecular self-assembly process. *Nat. Commun.* **8**, 14463 - 14471 (2017)
2. Packwood, D. M., Han, P., & Hitosugi, T. State-space reduction and equivalence class sampling for a molecular self-assembly model. *R. Soc. Open. Sci.* **3**, 150681 – 150701 (2016)
3. Packwood, D. M. & Hitosugi, T. Rapid prediction of molecule arrangements on metal surfaces *via* Bayesian optimization. *Appl. Phys. Express.* **10**, 065502 – 065506 (2017)
